# EGFR-HIF1α signaling positively regulates the differentiation of IL-9 producing T helper cells

**DOI:** 10.1038/s41467-021-23042-x

**Published:** 2021-06-01

**Authors:** Suyasha Roy, Zaigham Abbas Rizvi, Alexander J. Clarke, Felicity Macdonald, Abhaydeep Pandey, Dietmar Martin Werner Zaiss, Anna Kathrina Simon, Amit Awasthi

**Affiliations:** 1 Immuno-biology Laboratory, Translational Health Science & Technology Institute, Faridabad, Haryana India; 2 Kennedy Institute of Rheumatology, University of Oxford, Oxford, UK; 3 Institute of Immunology and Infection Research, University of Edinburgh, Scotland, UK; 4 Translational Health Science & Technology Institute, Faridabad, Haryana India

**Keywords:** Immunology, Lymphocytes, T cells, CD4-positive T cells

## Abstract

Interleukin 9 (IL-9)-producing helper T (Th9) cells are essential for inducing anti-tumor immunity and inflammation in allergic and autoimmune diseases. Although transcription factors that are essential for Th9 cell differentiation have been identified, other signaling pathways that are required for their generation and functions are yet to be explored. Here, we identify that Epidermal Growth Factor Receptor (EGFR) is essential for IL-9 induction in helper T (Th) cells. Moreover, amphiregulin (Areg), an EGFR ligand, is critical for the amplification of Th9 cells induced by TGF-β1 and IL-4. Furthermore, our data show that Areg-EGFR signaling induces HIF1α, which binds and transactivates IL-9 and NOS2 promoters in Th9 cells. Loss of EGFR or HIF1α abrogates Th9 cell differentiation and suppresses their anti-tumor functions. Moreover, in line with its reliance on HIF1α expression, metabolomics profiling of Th9 cells revealed that Succinate, a TCA cycle metabolite, promotes Th9 cell differentiation and Th9 cell-mediated tumor regression.

## Abstract (web summary)

Interleukin 9 (IL-9) producing helper T cells (Th) have established roles in a range of inflammatory conditions and a number of transcription factors have been shown crucial for the generation of these IL-9 producing cells. Here, the authors establish a role for the EGFR-HIF1α axis in the regulation of IL-9 producing Th cell populations.

## Introduction

IL-9, a pleiotropic cytokine of the common γ-chain family, was initially identified as a Th2 cytokine until it was defined in 2008 that IL-9 is exclusively produced by a distinct subset of helper T (Th) cells named as “Th9”. The differentiation of Th9 cells is induced primarily by transforming growth factor β1 (TGF-β1) and IL-4^1,2^. In fact, TGF-β1 can reprogram Th2 cells into Th9 cells^3–6^ as IL-4 signaling triggers Th9 cell differentiation by inhibiting TGF-β-mediated expression of Foxp3^1^. In addition to TGF-β1 and IL-4, cytokines such as IL-1α, IL-1β, IL-25, IL-33, and thymic stromal lymphopoietin (TSLP) have been shown to enhance IL-9 in Th9 cells^7–11^. Other CD4^+^ T cell subsets such as Th2, Th17, and iTregs are also known to produce IL-9, however, in lesser amounts than produced by Th9 cells^12–16^. Although cytokines and transcription factors, which initiate the process of differentiation of Th9 cells are known, the role of metabolic reprogramming in the generation and functions of Th9 cells is still not completely understood. Transcription factors that are downstream to TGF-β1, IL-4, and IL-2 signaling are essential for Th9 cell differentiation, as the deficiency of either of TGF-β receptor (TGF-βR), IL-4R or IL-2R impairs Th9 cell differentiation^1–3,17^. Transcription factors such as PU.1 (*Spi1)*, IRF4, BATF, GATA3, IRF1, and HIF1α, are found to play an essential role in the differentiation and functions of Th9 cells^18–23^.

Th9 cells are found to exacerbate allergic airway inflammation in asthma, colitis and eliminate helminth infections^24–26^. Importantly, Th9 cells possess potent anti-tumor functions particularly against melanoma and lung adenocarcinoma^27,28^. Consistently, *IL-9R*^−/−^mice or antibody mediated neutralization of IL-9 showed enhanced tumor progression while adoptive transfer of Th9 cells ameliorated tumor development in B16F10 melanoma and LLC-1 (Lewis Lung Carcinoma)^29^. Given the physiological importance of IL-9, particularly in anti-tumor immunity, a detailed understanding of molecular regulation of IL-9 induction in Th cells is needed.

Epidermal growth factor receptor (EGFR) is a member of the ErbB family and has been shown to express on epithelial and immune cells^30^. EGFR is activated upon the binding of its cognate ligands leading to the phosphorylation of its tyrosine kinase domain. Upon phosphorylation, downstream signaling pathways such as PI3K/AKT and RAS/MAPK are activated leading to cell proliferation, differentiation, and survival^30^. Among other EGFR ligands, Areg is found to be produced by Th2 cells and is important for helminth expulsion^31^. In addition, EGFR is expressed on Foxp3^+^ Tregs, and Areg-EGFR signaling is essential for the suppressive function of Tregs^32^. EGFR programs Th2 cells to function in a TCR independent fashion^33^. However, the role of Areg-EGFR axis in Th9 cells remains obscure.

EGFR signaling leads to HIF1α activation as shown in pancreatic cancer where EGFR maintains glucose metabolism through the activation of ERK1/2 pathway^34^. HIF1α plays an essential role in the differentiation and functions of Th cells^35^. HIF1α acts as one of the key metabolic checkpoints in differentiation and functions of Th9, Th17, and Tregs^23,36–38^. HIF1α regulates the expression of glycolytic genes and metabolic reprogramming of T cells from oxidative phosphorylation (OxPhos) to aerobic glycolysis^39^. We and others have shown that HIF1α increases the glycolytic activity in both mouse and human Th9 cells^23,38^. However, the regulation of IL-9 induction and metabolic pathways other than glycolysis, by HIF1α in Th cells has not been deciphered yet. Moreover, the crosstalk between EGFR and HIF1α has not been studied in context of IL-9 induction in Th9 cells.

Here, we show a positive regulation of IL-9 induction in Th cells by the EGFR-HIF1α signaling axis. We delineate a comprehensive network of IL-9 regulation in Th cells by the interactions among different micro-environmental cues and metabolites with the EGFR-HIF1α signaling cascade and its potential implications in anti-tumor immunity.

## Results

### EGFR signaling is essential for Th9 cell differentiation

**Fig. 1:**
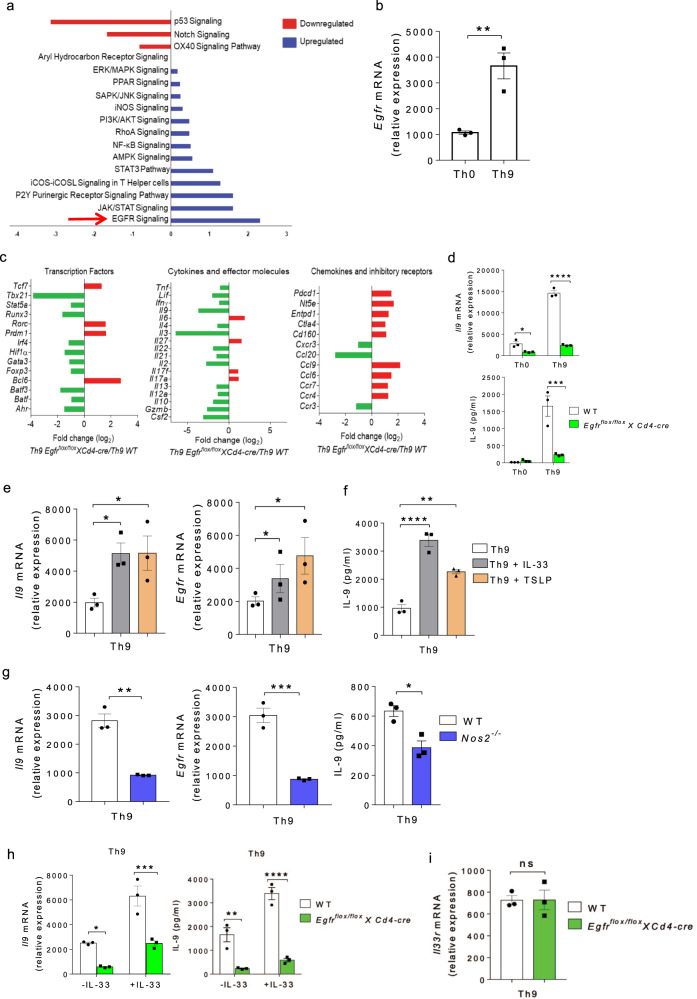
EGFR pathway is essential for Th9 cell differentiation. **a** Naïve CD4^+^ T cells from WT mice were in vitro differentiated into Th0 and Th9 followed by RNA-Seq and ingenuity pathway analysis. **b** qPCR analysis of *Egfr* expression. Data are representative of mean ± SEM from three independent experiments. **c**, **d** Naïve CD4^+^ T cells from WT and *Egfr*^*flox/flox*^*XCd4-cre* mice were in vitro differentiated under Th0 and Th9 polarizing conditions for 3 days followed by **c**. NanoString analysis. Fold change in relative expression relative to control was determined by log_2_ (Th9 *Egfr*^*flox/flox*^*XCd4-cre*/Th9 WT). **d** qPCR analysis of *Il9* expression and ELISA for IL-9 production. Data are representative of mean ± SEM from three independent experiments. **e**, **f** Naïve CD4^+^ T cells from WT mice were in vitro differentiated under Th9 polarizing conditions with or without 10 ng/ml IL-33 or 10 ng/ml TSLP for 3 days respectively; **e** qPCR analysis of *Il9, Egfr* expression and **f** ELISA for IL-9. Data are representative of mean ± SEM from three independent experiments. **g** Naïve CD4^+^ T cells from WT and *Nos2*^*−/−*^ mice were differentiated under Th9 polarizing conditions followed by qPCR analysis of *Il9* and *Egfr* expression and ELISA for IL-9. Data are representative of mean ± SEM from three independent experiments. **h** Naïve CD4^+^ T cells from WT and *Egfr*^*flox/flox*^*XCd4-cre* mice were differentiated under Th9 polarizing conditions with or without 10 ng/ml IL-33 for 3 days; qPCR analysis of *Il9* expression and ELISA for IL-9. Data are representative of mean ± SEM from three independent experiments. **i** Naïve CD4^+^ T cells from WT and *Egfr*^*flox/flox*^*XCd4-cre* mice were differentiated to Th9 followed by qPCR analysis of *Il33r* expression. Data are representative of mean ± SEM from three independent experiments. **b** **P = 0.007, using two-tailed unpaired Student’s *t* test. **d** *P = 0.033, ****P < 0.00001, ***P = 0.0007, using two-way ANOVA followed by Tukey’s multiple comparison test. **e**, **f** *P = 0.04, **P = 0.002, ****P < 0.0001, using one-way ANOVA followed by Tukey’s multiple comparison test. **g** **P = 0.003, ***P = 0.0009, *P = 0.01, using two-tailed unpaired Student’s *t* test. **h** *P = 0.05, ***P = 0.0009, **P = 0.001, ****P = 0.0001, using two-way ANOVA followed by Tukey’s multiple comparison test. **i** *P* = ns (not significant), using two-tailed unpaired Student’s *t* test.

We and others have previously identified that TGF-β1 together with IL-4 differentiate naive CD4^+^ T cells into IL-9-producing Th9 cells^1,2^. Since Th9 cells are critically involved in mounting a robust anti-tumor immune response^27,28^, we were keen to identify molecular pathways that lead to the induction of Th9 cells. To do this, naïve CD4^+^ T cells from wild-type (WT) mice were sorted as shown (Supplementary Fig. 1a), and differentiated into Th9 cells for performing global gene profiling followed by pathways analysis. Our RNA-Seq data analysis revealed different signaling pathways which were upregulated and downregulated in Th9 as compared to Th0 (naïve CD4^+^ T cells cultured without any cytokines). Among pathways that are upregulated in Th9 cells, the EGFR pathway was significantly enriched in Th9 as compared to Th0 cells (Fig. 1a), indicating the involvement of EGFR signaling in Th9 cell differentiation. In agreement with our RNA-Seq data, qPCR data also suggests that *Egfr* is differentially expressed in Th9 as compared to Th0 cells (Fig. 1b). To substantiate our claim, we used *Egfr*^*flox/flox*^*XCd4-cre* mice in which *Egfr* gene was conditionally deleted in CD4^+^ T cells. NanoString analysis revealed that, as compared to WT mice, Th9 cells from *Egfr*^*flox/flox*^*XCd4-cre* mice showed downregulation of key transcription factors, cytokines and chemokines that are known to be associated with Th9 cells while an upregulation of inhibitory receptors, suggesting that EGFR is essential for the developmental programming of Th9 cells (Fig. 1c). Consistently, there was a reduction in *Il9* expression and IL-9 production in Th9 cells from *Egfr*^*flox/flox*^*XCd4-cre* mice, as compared to WT Th9 cells (Fig. 1d).

Factors such as IL-33, TSLP and NO have been shown to enhance the generation of Th9 cells induced by TGF-β1 and IL-4^11,40,41^. However, whether these Th9-enhancing factors influence EGFR expression in Th9 cells is not known yet. We found that in the presence of IL-33 and TSLP, IL-9 and EGFR induction was amplified in Th9 cells (Fig. 1e, f). Further, we used the *Nos2*^−/−^mice to test the effect of NO on *Egfr* expression, and found that *Nos2*^−/−^ Th9 cells showed reduction in IL-9 and EGFR induction as compared to WT Th9 cells (Fig. 1g), suggesting an association of EGFR with IL-9 in Th9 cells. However, IL-33 was unable to completely restore IL-9 induction in Th9 cells in the absence of EGFR from *Egfr*^*flox/flox*^*XCd4-cre* mice as compared to WT Th9 cells (Fig. 1h). It could be possible that IL-33 receptor (IL-33R), in turn, is regulated by EGFR in Th9 cells. To rule this out, we tested the expression of IL-33R in WT and EGFR-deficient Th9 cells, and did not observe any change in the expression of *Il33r* in EGFR-deficient Th9 cells as compared to WT Th9 cells (Fig. 1i). These data indicates that IL-33-induced enhancement of IL-9 in Th9 cells is mediated through EGFR signaling.

### Inhibition of EGFR signaling abrogates anti-tumor functions of Th9 cells

**Fig. 2:**
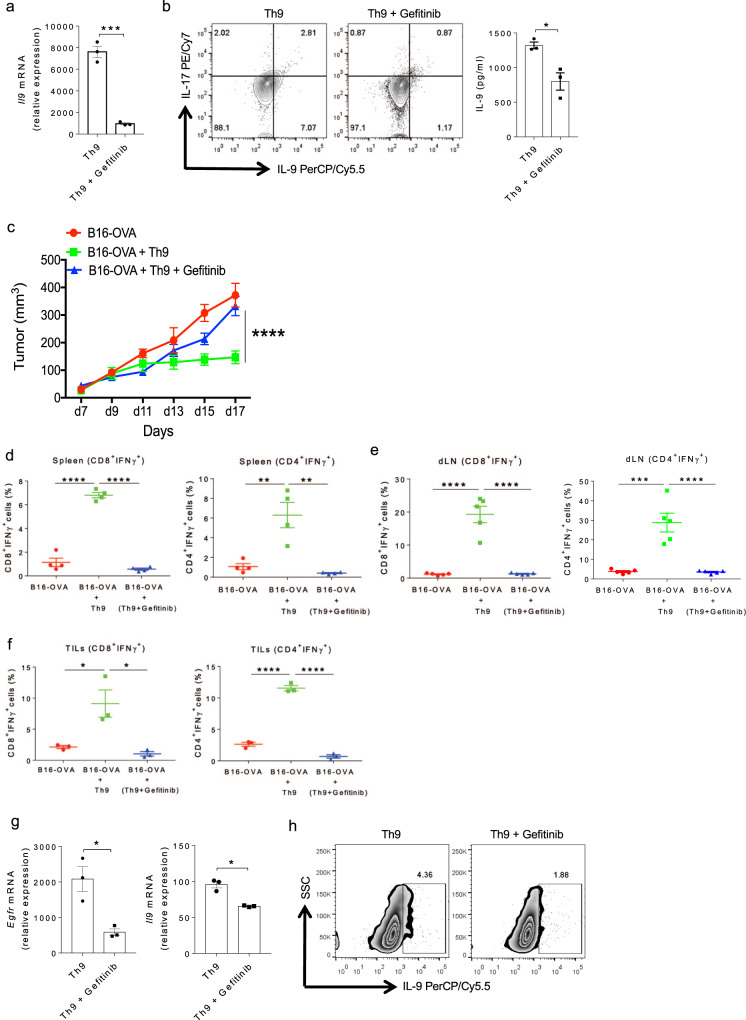
EGFR inhibition abrogates the anti-tumor functions of Th9 cells. **a**, **b** Naïve CD4^+^ T cells from WT mice were in vitro differentiated under Th9 conditions with or without 1.0 μM gefitinib for 3 days followed by **a**. qPCR analysis of *Il9* expression. **b** ELISA for IL-9 and flow cytometry analysis of intracellular staining for IL-9 and IL-17. Data are representative of mean ± SEM from three independent experiments. **c**–**f** Naïve CD4^+^ T cells from OT-II TCR transgenic mice were in vitro differentiated into Th9 with or without 1.0 μM gefitinib for 3 days. Cells were then adoptively transferred into B16-OVA tumor-bearing WT mice, randomized into three groups (*n* = 5 mice per group). **c** Mean tumor volume was measured over time shown as tumor growth curve. **d**, **e** Spleen and tumor draining lymph nodes (dLN) were harvested and single cell suspensions were made followed by FACS analysis of intracellular staining for CD4^+^IFNγ^+^ and CD8^+^IFNγ^+^. **f** TILs were isolated from the tumor followed by FACS analysis of intracellular staining for CD8^+^IFNγ^+^and CD4^+^IFNγ^+^ cell populations. Data are representative of mean ± SEM from three independent experiments. **g**, **h** Sorted naïve human CD4^+^ T cells were differentiated into Th9 cells with or without 1.0 μM gefitinib. **g** mRNA expression of *Egfr* and *Il9* was determined by qPCR. Data are representative of mean ± SEM from three healthy individuals. **h** Intracellular staining for IL-9. **a**, **b** ***P = 0.0003, *P = 0.017, using two-tailed unpaired Student’s *t* test. **c** ****P < 0.0001, using two-way ANOVA followed by Tukey’s multiple comparison test. **d**–**f** ****P < 0.0001, **P = 0.001, ***P = 0.0001, *P = 0.02, using one-way ANOVA followed by Tukey’s multiple comparison test. **g** *P = 0.014, using two-tailed unpaired Student’s *t* test.

Anti-tumor functions of Th9 cells have been clearly demonstrated in melanoma^28^. Our data indicates that EGFR signaling is essential for the differentiation of Th9 cells. To functionally validate the role of EGFR signaling in Th9 cells, we inhibited EGFR functions in Th9 cells using gefitinib, which inhibits the tyrosine kinase activity of EGFR by binding to its ATP-binding domain. Naïve CD4^+^ T cells isolated from WT mice were differentiated into Th9 cells in the absence or presence of gefitinib to further test the effect of EGFR inhibition on Th9 cells. Blocking EGFR signaling by gefitinib significantly inhibited the *Il9* expression and IL-9 production in Th9 cells (Fig. 2a, b). To test the in vivo effect of EGFR inhibition, naïve CD4^+^ T cells from OT-II TCR transgenic mice were in vitro polarized into Th9 cells in the presence or absence of gefitinib. These cells were then adoptively transferred into a B16-OVA tumor-bearing WT mice and the tumor progression was monitored. Th9 cells significantly regressed tumor growth while the anti-tumor functions of Th9 cells was abrogated in the presence of gefitinib (Fig. 2c). Further, we found a decrease in the frequency of IFN-γ producing CD8^+^ and CD4^+^ T lymphocytes in the spleen as well as in the tumor draining lymph nodes (dLN) in the group of mice transferred with gefitinib-treated OT-II Th9 cells as compared to OT-II-Th9 cells (Fig. 2d, e and Supplementary Fig. 2a–f). Frequencies of both CD8^+^ IFN-γ^+^ and CD4^+^ IFN-γ^+^ tumor-infiltrating lymphocytes (TILs) were also reduced within the gefitinib-treated group (Fig. 2f and Supplementary Fig. 2g, h). These data suggests that EGFR signaling is required for the anti-tumor functions of Th9 cells. Next, sorted human naïve CD4^+^ T cells (Supplementary Fig. 1b) were differentiated into Th9 cells with or without gefitinib. Likewise, blocking EGFR signaling by gefitinib suppressed EGFR and IL-9 induction in human Th9 cells also (Fig. 2g, h). Taken together, these data emphasizes that EGFR signaling is essential for Th9 cell differentiation in both mouse and humans.

Since IL-9 is known to be also produced by Th2, Th17, and Tregs although in lesser amounts^15,16,18^, so we next tested the effect of EGFR inhibition on IL-9 induction in Th2, Th17, and iTregs. We found that blocking EGFR signaling using gefitinib significantly suppressed the IL-9 induction in Th2, Th17, and iTregs (Supplementary Fig. 3a). In addition to IL-9, in vitro differentiated Th9 cells also produce IL-10^1^ while Th9 cells eliminate tumor in vivo by triggering IFN-γ production by CD8^+^ T cells^22^. To understand whether EGFR-deficient Th9 cells tend to produce IL-10 and/or IFN-γ and have the potential to adopt different phenotypes, WT and EGFR-deficient CD4^+^ T cells were differentiated into Th9 cells and the levels of IL-10 and IFN-γ were determined. There were no significant changes observed in the induction of both IL-10 and IFN-γ in EGFR-deficient Th9 cells as compared to WT Th9 cells (Supplementary Fig. 3b, c). These results apparently, indicates that the abrogation of EGFR signaling repressed IL-9 induction without influencing the expression of other cytokines in Th9 cells.

### Areg is required for EGFR-mediated IL-9 induction in Th cells

**Fig. 3:**
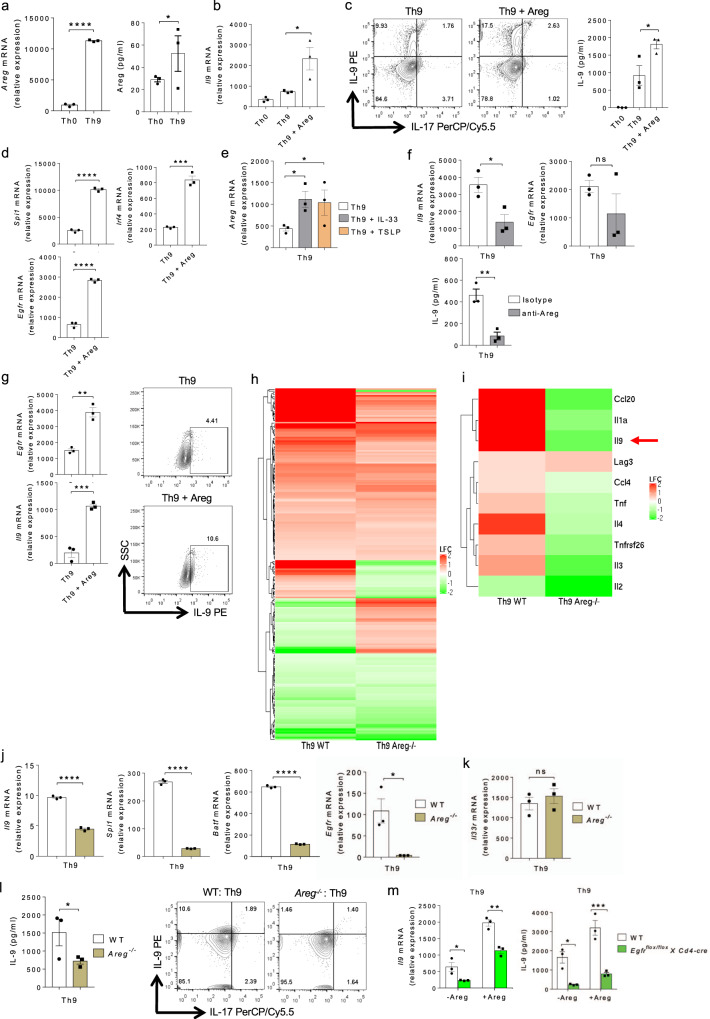
Areg promotes EGFR-mediated Th9 cell differentiation. **a** Naïve CD4^+^ T cells from WT mice were differentiated into Th0 and Th9 followed by qPCR analysis and ELISA for Areg. Data are representative of mean ± SEM from three independent experiments. **b**–**d** Naïve CD4^+^ T cells from WT mice were differentiated into Th0 and Th9 with or without 100 ng/ml Areg followed by **b** qPCR analysis of *Il9* expression. **c** FACS staining for IL-9 and IL-17 and ELISA for IL-9. **d** qPCR for *Spi1*, *Irf4*, and *Egfr* expression. Data are representative of mean ± SEM from three independent experiments. **e** Naïve CD4^+^ T cells from WT mice were differentiated under Th9 polarizing conditions with or without 10 ng/ml IL-33 or 10 ng/ml TSLP respectively for 3 days followed by qPCR analysis of *Areg* expression. Data are representative of mean ± SEM from three independent experiments. **f** Naïve CD4^+^ T cells from WT mice were differentiated into Th9 in the presence of isotype antibody (Ab) or anti-Areg Ab followed by qPCR analysis of *Il9, Egfr* expression and ELISA for IL-9. Data are representative of mean ± SEM from three independent experiments. **g** Sorted naïve human CD4^+^ T cells were differentiated into Th9 cells with or without 100 ng/ml Areg. qPCR analysis of *Il9* and *Egfr* expression and FACS for IL-9. Data are representative of mean ± SEM from three healthy individuals. **h**–**l** Naïve CD4^+^ T cells from WT and *Areg*^*−/−*^ mice were differentiated under Th9 polarizing conditions. **h** Heat-map of significantly differentially expressed genes in Th9 cells from WT and *Areg*^*−/−*^ mice after RNA-Seq analysis. **i** Heat-map for RNA-Seq analysis of selected significantly differentially expressed genes in Th9 cells from WT and *Areg*^*−/−*^ mice. **j**, **k** qPCR analysis of *Il9, Spi1*, *Batf*, *Egfr*, and *Il33r* expression. **l** ELISA for IL-9 and flow cytometry analysis of intracellular staining for IL-9. Data are representative of mean ± SEM from three independent experiments. **m** Naïve CD4^+^ T cells from WT and *Egfr*^*flox/flox*^*XCd4-cre* mice were differentiated under Th9 polarizing conditions with or without 100 ng/ml Areg for 3 days; qPCR analysis of *Il9* expression and ELISA for IL-9. Data are representative of mean ± SEM from three independent experiments. **a** ****P < 0.0001, *P = 0.04, using two-tailed unpaired Student’s *t* test. **b** *P = 0.02, using one-way ANOVA followed by Tukey’s multiple comparison test. **c** *P = 0.02 using one-way ANOVA followed by Tukey’s multiple comparison test. **d** ****P < 0.0001, ***P = 0.0003, using two-tailed unpaired Student’s *t* test. **e** *P = 0.01, using one-way ANOVA followed by Tukey’s multiple comparison test. **f** *P = 0.01, *P* = ns (not significant), **P = 0.005, using two-tailed unpaired Student’s *t* test. **g** **P = 0.0044, ***P = 0.0006, using two-tailed unpaired Student’s *t* test. **j**, **k** ****P < 0.0001, *P = 0.02, *P* = ns (not significant), using two-tailed unpaired Student’s *t* test. **l** *P = 0.04, using two-tailed unpaired Student’s *t* test. **m** *P = 0.01, **P = 0.001, ***P = 0.0006, using two-way ANOVA followed by Tukey’s multiple comparison test.

Since EGFR activation requires the binding of its ligands, therefore we tested the expression of different EGFR ligands in Th9 cells, and found that Areg was upregulated both at mRNA and protein level in Th9 cells as compared to Th0 cells (Fig. 3a). Other EGFR ligands such as *Tgfα*, *Egf*, and *Begf* were not found to be expressed in Th9 cells as compared to Th0, suggesting a potential role of Areg-EGFR axis in the differentiation and functions of Th9 cells (Supplementary Fig. 4a). Interestingly, Th2, Th17, and iTregs expressed *Areg* and *Egfr,* in addition to *Il9,* at lower levels as compared to Th9 cells, indicating a positive correlation between Areg and IL-9 induction in Th cells (Supplementary Fig. 4b). To further validate the role of Areg in Th9 cell differentiation, we polarized Th9 cells in vitro with or without exogenous Areg. In the presence of exogenous Areg, the expression of *Il9* and IL-9 production increased significantly in Th9 cells (Fig. 3b, c). In addition, the expression of Th9-associated genes, *Spi1* and *Irf4*, was increased in Th9 cells cultured with exogenous Areg as compared to Th9 cells cultured without exogenous Areg (Fig. 3d). Moreover, exogenous Areg also enhanced *Egfr* expression in Th9 cells as compared to Th9 cells cultured without exogenous Areg (Fig. 3d). Consistently, Areg also boosted IL-9 and EGFR induction in Th2, Th17, and iTregs (Supplementary Fig. 4c). Furthermore, Th9-enhancing factors such as IL-33^10,40^ and TSLP^11^, led to an enhanced *Areg* expression in Th9 cells (Fig. 3e). In line with these observations, Areg neutralization with anti-Areg antibody significantly abrogated *Il9* and *Egfr* expression and IL-9 production in Th9 cells (Fig. 3f). Similar to the findings in mouse Th9 cells, supplementation of exogenous Areg also increased EGFR and IL-9 induction in human Th9 cells (Fig. 3g).

To further substantiate our claim on the role of Areg in Th9 cell differentiation, we isolated naïve CD4^+^ T cells from WT and *Areg*^−/−^ mice and differentiated them into Th9 cells. Transcriptomics profiling identified differentially expressed genes in *Areg*^−/−^ Th9 cells as compared to WT Th9 cells (Fig. 3h). *Il9* was identified among the top downregulated genes in *Areg*^−/−^ Th9 cells as compared to WT Th9 cells (Fig. 3i). We validated our RNA-Seq data by qPCR, and found that the expression of *Il9* and Th9 cell-associated transcription factors, *Spi1* and *Batf*, were decreased in *Areg*^−/−^ Th9 cells as compared to WT Th9 cells (Fig. 3j). In addition, as compared to WT Th9 cells, *Areg*^−/−^ Th9 cells showed a reduced expression of *Egfr* without affecting the expression of *Il33r* (Fig. 3j, k), suggesting that Areg is essential for EGFR and IL-9 induction in Th9 cells. There was also diminished IL-9 production in *Areg*^−/−^ Th9 cells as compared to WT Th9 cells (Fig. 3l). Apparently, there was diminished IL-9 induction and *Egfr* expression in Th2, Th17, and iTregs upon *Areg* deletion (Supplementary Fig. 4d). While exogenous Areg failed to enhance *Il9* expression in Th0 cells (Supplementary Fig. 4e), exogenous Areg together with TGF-β1 plus IL-4 resulted in a higher *Il9* expression as compared to TGF-β1 or IL-4 alone (Supplementary Fig. 4f). Strikingly, in *Egfr*^*flox/flox*^*XCd4-cre* mice, Areg could not completely restore IL-9 induction in Th9 cells as compared to WT mice (Fig. 3m), indicating the fact that Areg mediates its effect on the differentiation of Th9 cells via EGFR. We further tested whether Areg-deficient Th9 cells produce IL-10 and IFN-γ, as these cytokines were found to be produced by Th9 cells in vitro and in vivo respectively. Our data indicates that there were no differential induction of IL-10 and IFN-γ in Areg-deficient Th9 cells, as compared to WT Th9 cells (Supplementary Fig. 4g, h). Taken together, these data demonstrated that Areg-mediated EGFR activation amplifies IL-9 induction in IL-9 producing Th cells.

### HIF1α is critical for IL-9 induction in Th cells

**Fig. 4:**
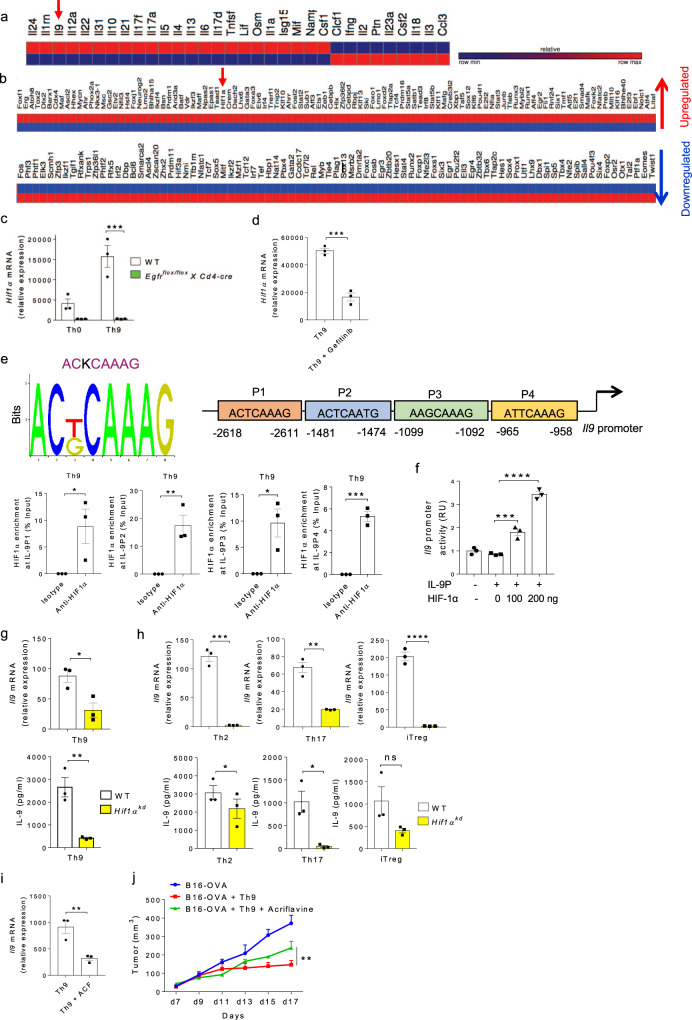
HIF1α is critical for IL-9 induction in Th cells. **a**, **b** Heat-map for RNA-Seq analysis of significantly differentially expressed genes in Th9 cells as compared to Th0 cells. **c** Naïve CD4^+^ T cells from WT and *Egfr*^*flox/flox*^*XCd4-cre* mice were differentiated under Th0 and Th9 polarizing conditions for 3 days followed by qPCR analysis of *Hif1α* expression. Data are representative of mean ± SEM from three independent experiments. **d** Naïve CD4^+^ T cells from WT mice were differentiated under Th9 polarizing conditions with or without 1.0 μM gefitinib for 3 days followed by qPCR analysis of *Hif1α* expression. Data are representative of mean ± SEM from three independent experiments. **e** Bioinformatics analysis of HIF1α binding motif in IL-9 promoter. ChIP analysis of HIF1α binding to IL-9 promoter in Th9 cells represented as enrichment of HIF1α on IL-9 promoter relative to input. Data are representative of mean ± SEM from three independent experiments. **f** Luciferase reporter assay for IL-9 promoter activity in the presence of HIF1α plasmid at 0, 100, and 200 ng concentrations. Data are representative of mean ± SEM from three independent experiments. **g**, **h** Naïve CD4^+^ T cells from WT and *Hif1α*^*kd*^ mice were differentiated under Th9, Th2, Th17, iTregs polarizing conditions with daily treatment of 1.0 μg/ml Dox for 3 days followed by qPCR analysis of *Il9* expression and ELISA for IL-9 production. Data are representative of mean ± SEM from three independent experiments. **i** Naïve CD4^+^ T cells from WT mice were differentiated into Th9 cells with or without 5.0 μM acriflavine (ACF) followed by qPCR analysis of *Il9* expression. **j** Naïve CD4^+^ T cells from OT-II TCR transgenic mice were differentiated into Th9 cells with or without 5.0 μM acriflavine (ACF). At day 4, cells were adoptively transferred into B16-OVA tumor-bearing WT mice, randomized into three groups. Mean tumor volume was measured over time. Data are representative of mean ± SEM from three independent experiments, (*n* = 5 mice per group). **c** ***P = 0.0004, using two-way ANOVA followed by Tukey’s multiple comparison test. **d** ***P = 0.0006, using two-tailed unpaired Student’s *t* test. **e** *P = 0.04, **P = 0.009, ***P = 0.0003, using two-tailed unpaired Student’s *t* test. **f** ***P = 0.0009, ****P < 0.0001, using one-way ANOVA followed by Tukey’s multiple comparison test. **g**, **h** *P = 0.02, **P = 0.0027, ***P = 0.0002, ****P < 0.0001, *P* = ns (not significant), using two-tailed unpaired Student’s *t* test. **i** **P = 0.01, using two-tailed unpaired Student’s *t* test. **j** **P = 0.0014, using two-way ANOVA followed by Tukey’s multiple comparison test.

Transcription factor HIF1, composed of HIF1α and HIF1β subunits, is involved predominantly in controlling the differentiation and functions of Th cells^35^. We have previously reported the impact of HIF1α on IL-9 induction in human Th9 cells^38^, and it is known that HIF1α promotes mouse Th9 cell differentiation^23^. Our RNA-Seq analysis identified an upregulation of *Il9* and *Hif1α* genes expression in Th9 cells (Fig. 4a, b). Among other transcription factors, HIF1α was found to be a major transcription factor that is crucial for metabolic regulation of T cell differentiation. Considering the role of HIF1α in influencing the metabolomic regulation in T cells differentiation, we picked HIF1α to identify its role at molecular level in the induction of IL-9 in Th cells. Moreover, it has been demonstrated that Areg-EGFR signaling merges to HIF1α, and consistently our NanoString data (Fig. 1c, left most panel) indicated that the expression of HIF1α was substantially downregulated in EGFR-deficient Th9 cells as compared to WT Th9 cells, which led us to focus on HIF1α in order to identify the significance of Areg-EGFR-HIF1α axis in the differentiation and function of Th9 cells.

To elucidate the downstream signaling cascade of EGFR pathway, we sought to investigate the role of EGFR-HIF1α axis in IL-9 induction and whether Areg-EGFR axis converge to HIF1α during Th9 cell differentiation. We found that Th9 cells from *Egfr*^*flox/flox*^*XCd4-cre* mice failed to express *Hif1α* as compared to WT Th9 cells (Fig. 4c). Moreover, inhibition of EGFR signaling by gefitinib resulted in the abrogation of *Hif1α* expression while in the presence of exogenous Areg, *Hif1α* expression was remarkably increased (Fig. 4d and Supplementary Fig. 5a), suggesting that Areg-EGFR pathway induces HIF1α in Th9 cells.

To demonstrate the functional association of HIF1α with IL-9, we identified four putative HIF1α-binding sites in the proximal promoter of IL-9. We performed the chromatin immunoprecipitation (ChIP) assay to confirm the physical binding of HIF1α to IL-9 promoter, and found that HIF1α binds to all the four putative HIF1α-binding sites on IL-9 promoter in Th9 cells (Fig. 4e). To further establish the functionality of HIF1α binding to IL-9 promoter, we performed luciferase reporter assay to measure IL-9-promoter driven-luciferase activity. Our data confirmed that HIF1α transactivates IL-9 promoter activity resulting in increased *Il9* transcription (Fig. 4f). In addition to HIF1α binding to IL-9 promoter in Th9 cells, our ChIP data also confirmed that HIF1α binds to IL-9 promoter in Th2, Th17 cells as well as iTregs, since these cells tend to produce lower amounts of IL-9, as compared to Th9 cells (Supplementary Fig. 5b–d).

The physiological role of HIF1α in IL-9 induction was validated by knocking down *Hif1α* gene in CD4^+^ T cells using *Hif1α*^*kd*^ mice in which shRNA silences *Hif1α* expression upon doxycycline (Dox) induction. Naïve CD4^+^ T cells, isolated from WT and *Hif1α*^*kd*^ mice, were in vitro differentiated into Th9, Th2, Th17, and iTregs with daily treatment of 1.0 μg/ml Dox for 3 days. qPCR and ELISA results showed that *Il9* expression was decreased and IL-9 secretion was also dampened in Th9 cells differentiated from *Hif1α*^*kd*^ mice as compared to WT mice (Fig. 4g). Consistently, it was observed that the other IL-9-producing T cells such as Th2, Th17 cells and iTregs from *Hif1α*^*kd*^ mice as compared to WT mice, have shown reduced IL-9 induction at both mRNA and protein levels (Fig. 4h), suggesting that HIF1α is essentially required for IL-9 induction in Th cells. As indicated earlier, we did not find any substantial difference in the induction of cytokines, IL-10 and IFN-γ when *Hif1α* was knocked down in Th9 cells (Supplementary Fig. 5e, f). To test the physiological relevance of HIF1α expression in Th9 cells, we performed Th9 cells adoptive transfer experiments in B16-OVA melanoma model, and observed that the anti-tumor activity of Th9 cells was abrogated in the presence of acriflavine (ACF), a HIF1α inhibitor^42^ (Fig. 4i, j). Taken together, these results clearly demonstrates the role of EGFR-HIF1α axis in the induction and functions of Th9 cells.

### PHD2 and Hypoxia-mediated HIF1α stabilization and IL-9 induction in Th cells

**Fig. 5:**
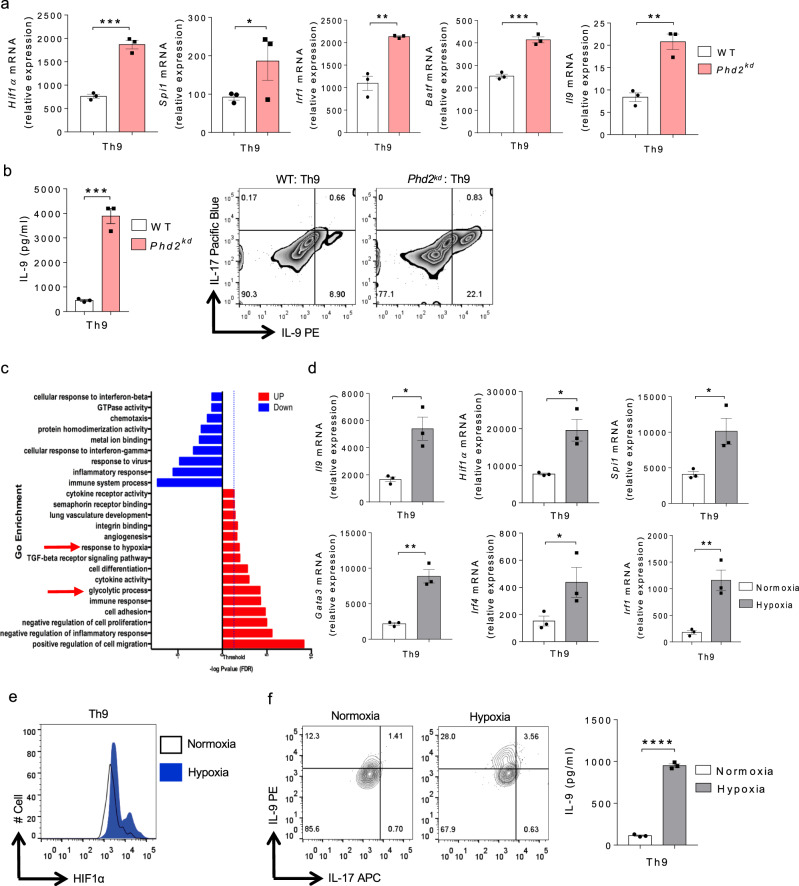
PHD2 and Hypoxia-mediated HIF1α stabilization and IL-9 induction in Th cells. **a**, **b** Naïve CD4^+^ T cells from WT and *Phd2*^*kd*^ mice were differentiated under Th9 polarizing conditions with daily treatment of 1.0 μg/ml Dox for 3 days. **a** qPCR analysis of *Hif1α, Spi1, Irf1*, *Batf* and *Il9 *expression. Data are representative of mean ± SEM from three independent experiments. **b** ELISA and FACS for IL-9 production. Data are representative of mean ± SEM from three independent experiments. **c** Pathway analysis depicting the enrichment of pathways associated with HIF1α in WT Th9 cells. **d**–**f** Naïve CD4^+^ T cells from WT mice were differentiated into Th9 under normoxic (21% oxygen) or hypoxic (1.0% oxygen) conditions for 3 days. **d** qPCR analysis of *Il9*, *Hif1α*, *Spi1*, *Gata3*, *Irf4*, and *Irf1* expression. Data are representative of mean ± SEM from three independent experiments. **e** FACS staining for HIF1α. **f** IL-9 production by flow cytometry and ELISA. Data are representative of mean ± SEM from three independent experiments. **a**, **b** ***P = 0.0004, *P = 0.04, **P = 0.0018, using two-tailed unpaired Student’s *t* test. **d**, **f** *P = 0.02, **P = 0.007, ****P < 0.0001, using two-tailed unpaired Student’s *t* test.

Since we showed that EGFR-HIF1α axis is critical for IL-9 induction in Th cells, we next sought to investigate the role of regulators of HIF1α stability and its subsequent effect on IL-9 induction in Th cells. It has been reported that HIF1 and HIF2 is stable in the absence of prolyl hydroxylases 2 (PHD2)^43–46^. To test the role of PHD2 in IL-9 induction, we used Dox-inducible *Phd2*^*kd*^ mice^47^ in which *Phd2* gene encoding for prolyl hydroxylases 2 (PHD2) was knocked down upon Dox treatment. Naïve CD4^+^ T cells isolated from WT and *Phd2*^*kd*^ mice were differentiated under Th9 polarizing conditions with daily treatment of 1.0 μg/ml Dox for 3 days. qPCR analysis suggests an upregulation of *Hif1α* in Th9 cells from *Phd2*^*kd*^ mice as compared to WT mice (Fig. 5a). This is in accordance with the previous findings suggesting that *Hif1α* expression is higher and more stable in the absence of PHD2^ 43^. PHD2 regulates both HIF1 and HIF2 activation and consistently, *Il9* expression was found to be higher in *Phd2*^*kd*^ mice as compared to WT mice (Fig. 5a, right most panel). This could be due to the higher HIF stability triggering an enhanced *Il9* expression in Th9 cells in the absence of PHD2. Further, we checked Th9-associated genes through qPCR and found that *Spi1*, *Irf1*, and *Batf* were upregulated in Th9 cells from *Phd2*^*kd*^ mice as compared to WT mice (Fig. 5a). IL-9 production was also increased in Th9 cells from *Phd2*^*kd*^ mice as compared to WT mice (Fig. 5b). In addition, we found that PHD2 knockdown, using the *Phd2*^*kd*^ mice, significantly enhanced IL-9 induction at both mRNA and protein levels in Th2, Th17 and iTregs as well (Supplementary Fig. 6a), illustrating that knocking down *Phd2* gene promotes IL-9 induction in all Th cells.

In addition to the EGFR pathway, RNA-Seq re-analysis of mouse Th9 cells identified the “enrichment of glycolysis” and “response to hypoxia” pathways in Th9 cells (Fig. 5c). We have previously shown that HIF1α is stabilized and constitutively expressed in hypoxic condition which triggers human Th9 cell differentiation^38^. Corroborating our published human data^38^, mouse Th9 cells differentiated in hypoxic conditions showed an increase in the expression of *Il9*, *Hif1α*, and signature genes in Th9 cells (Fig. 5d). HIF1α protein stability was further enhanced in hypoxia in comparison to normoxia in Th9 cells (Fig. 5e). IL-9 production by Th9 cells was also increased in hypoxia (Fig. 5f). In addition, IL-9 induction was also enhanced in Th2, Th17, and iTregs differentiated under hypoxic conditions (Supplementary fig. 6b). All together, these data exemplifies that PHD2 and hypoxia stabilizes HIF1α boosting IL-9 induction in Th cells.

### NO and HIF1α synergistically triggers IL-9 induction in Th9 cells

**Fig. 6:**
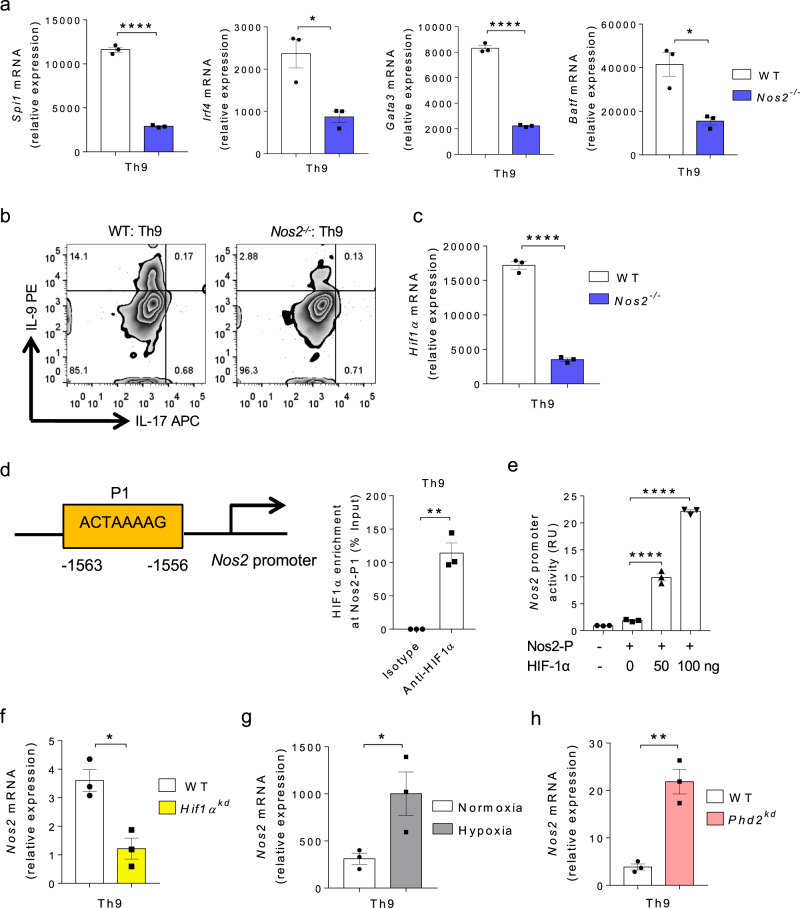
NO and HIF1α synergistically triggers IL-9 induction in Th9 cells. **a**–**c** Naïve CD4^+^ T cells from WT and *Nos2*^*−/−*^ mice were differentiated to Th9 cells followed by **a** qPCR analysis of *Spi1, Irf4, Gata3* and *Batf* expression. Data are representative of mean ± SEM from three independent experiments. **b** FACS staining for IL-9 and IL-17 in Th9 cells. **c** qPCR analysis of *Hif1α* expression. Data are representative of mean ± SEM from three independent experiments. **d** Bioinformatics analysis of HIF1α binding motif in Nos2 promoter followed by ChIP analysis of HIF1α binding to Nos2 promoter in Th9 cells represented as enrichment of HIF1α on Nos2 promoter relative to input. Data are representative of mean ± SEM from three independent experiments. **e** Luciferase assay for Nos2 promoter activity in the presence of HIF1α plasmid at 0, 50, and 100 ng concentrations. Data are representative of mean ± SEM from three independent experiments. **f** Naïve CD4^+^ T cells from WT and *Hif1α*^*kd*^ mice were differentiated under Th9 polarizing conditions with daily treatment of 1.0 μg/ml Dox for 3 days followed by qPCR analysis of *Nos2* expression. Data are representative of mean ± SEM from three independent experiments. **g** Naïve CD4^+^ T cells from WT mice were differentiated into Th9 cells under normoxic (21% oxygen) or hypoxic (1% oxygen) conditions for 3 days followed by qPCR analysis of *Nos2* expression. Data are representative of mean ± SEM from three independent experiments. **h** Naïve CD4^+^ T cells from WT and *Phd2*^*kd*^ mice were differentiated under Th9 polarizing conditions with daily treatment of 1.0 μg/ml Dox for 3 days followed by qPCR analysis of *Nos2* expression. Data are representative of mean ± SEM from three independent experiments. **a**, **c** ****P < 0.00001, *P = 0.01, using two-tailed unpaired Student’s *t* test. **d** **P = 0.0017, using two-tailed unpaired Student’s *t* test. **e** ****P < 0.00001, using one-way ANOVA followed by Tukey’s multiple comparison test. **f** *P = 0.01, using two-tailed unpaired Student’s *t* test. **g** *P = 0.04, using two-tailed unpaired Student’s *t* test. **h** **P = 0.0026, using two-tailed unpaired Student’s *t* test.

It is known that NO enhances Th9 cell differentiation^41^ and we found an association between NO and HIF1α in human Th9 cells^38^. Our data suggests that NO is required for EGFR expression in Th9 cells (Fig. 1g). However, the link between NO and HIF1α in context of IL-9 induction in mouse Th cells is not yet known. To test this hypothesis, we examined the effect of NO in Th9 cells using *Nos2*^−/−^ mice. We found that the expression of Th9-associated genes, *Spi1*, *Irf4*, *Gata3*, and *Batf* were downregulated in *Nos2*^−/−^ Th9 cells as compared to WT Th9 cells (Fig. 6a). Consistently, IL-9 production was also reduced in *Nos2*^−/−^ Th9 cells (Fig. 6b). However, there was no detectable difference in the induction of IL-10 and IFN-γ in *Nos2*^−/−^ Th9 cells as compared to WT Th9 cells (Supplementary Fig. 7a, b). In addition, IL-9 induction was also diminished in *Nos2*^−/−^ Th2, Th17, and iTregs, suggesting that NO is essential for IL-9 induction in all IL-9 producing Th cell subsets (Supplementary Fig. 7c). It has been shown that NO promotes HIF1α stabilization^48^ and we also found that the expression of *Hif1α* was inhibited in *Nos2*^−/−^ Th9 cells (Fig. 6c). Interestingly, ChIP and luciferase assays showed that HIF1α binds and transactivates *Nos2* promoter (Fig. 6d, e). This was further corroborated with the finding that *Hif1α*^*kd*^ Th9 cells express reduced *Nos2* expression in Th9 cells (Fig. 6f). Similarly, *Nos2* expression was higher in Th9 cells cultured in hypoxia as compared to normoxia (Fig. 6g). Consistently, *Nos2* expression was also elevated in Th9 cells from *Phd2*^*kd*^ mice as compared to WT mice (Fig. 6h), suggesting that increased HIF1α activity results in higher *Nos2* expression in Th9 cells. These data interprets that NO and HIF1α creates a feed-forward loop to promote Th9 cell differentiation synergistically.

### αKG and succinate reciprocally regulates HIF1α and IL-9 induction in Th9 cells

**Fig. 7:**
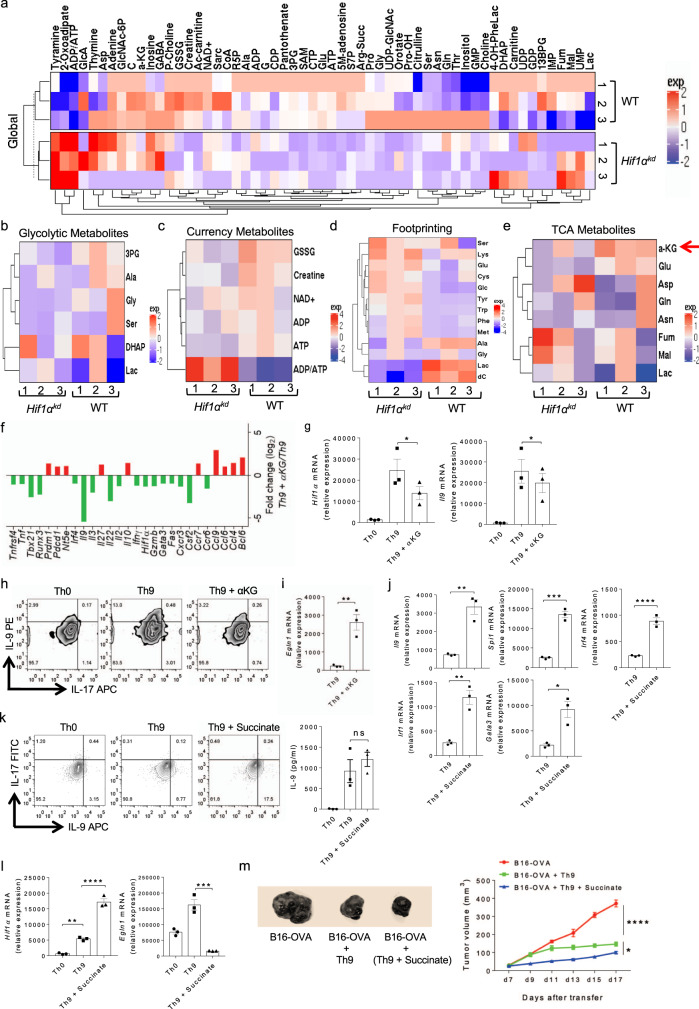
Succinate enhances HIF1α-mediated Th9 cell differentiation and anti-tumor immunity. **a**–**e** Naïve CD4^+^ T cells from WT and *Hif1α*^*kd*^ mice were differentiated under Th9 polarizing conditions. Samples were prepared and subjected to metabolomics. Heat-maps showing **a** global distribution and quantification of metabolites in the cell extracts; **b** differentially expressed metabolites of glycolytic pathway; **c** differentially expressed currency metabolites in cell extract; **d** footprinting quantification of differentially expressed metabolites in the cell culture supernatants, and **e** differentially expressed metabolites of TCA cycle in the cell extracts. **f** Naïve CD4^+^ T cells from WT mice were differentiated to Th0 and Th9 with or without 1.0 mM αKG, followed by NanoString analysis of mRNA expression in Th9 and Th9 + αKG conditions. Fold change in relative expression relative to control as determined by log_2_ (Th9 + αKG/Th9 WT). **g**–**i** Naïve CD4^+^ T cells from WT mice were differentiated to Th0 and Th9 with or without 1.0 mM αKG followed by **g** qPCR analysis of *Hif1α* and *Il9* expression. Data are representative of mean ± SEM from three independent experiments. **h** FACS analysis of intracellular IL-9 and IL-17 staining, and **i** mRNA expression of *Egln1*. Data are representative of mean ± SEM from three independent experiments. **j**–**l** Naïve CD4^+^ T cells from WT mice were differentiated into Th9 cells with or without 5.0 mM succinate followed by **j** qPCR analysis of *Il9*, *Spi1*, *Irf4*, *Irf1*, and *Gata3* expression. **k** FACS analysis of intracellular IL-9 and IL-17 production and ELISA for IL-9. **l** qPCR analysis of *Hif1α* and *Egln1* mRNA expression. Data are representative of mean ± SEM from three independent experiments. **m** Naïve CD4^+^ T cells from OT-II TCR transgenic mice were differentiated into Th9 cells with or without 5.0 mM succinate. At day 4, cells were adoptively transferred into B16-OVA tumor-bearing WT mice. Mean tumor volume was measured over time. Data represent one of the three experiments with three independently analyzed mice/group (*n* = 3 mice per group). **g** *P = 0.009, using one-way ANOVA followed by Tukey’s multiple comparison test. **i** **P = 0.004, using two-tailed unpaired Student’s *t* test. **j** **P = 0.0027, ***P = 0.0002, ****P < 0.0001, *P = 0.011, using two-tailed unpaired Student’s *t* test. **k**. *P* = ns (not significant), using one-way ANOVA followed by Tukey’s multiple comparison test. **l** **P = 0.0046, ****P < 0.0001, ***P = 0.0001, using one-way ANOVA followed by Tukey’s multiple comparison test. **m** ****P < 0.0001, *P = 0.033, using two-way ANOVA followed by Tukey’s multiple comparison test.

It is well known that metabolic regulation plays a key role in Th cell differentiation^49^, and our data indicates a critical role of HIF1α in Th9 cell differentiation. Since HIF1α is one of the known transcription factor that regulate metabolic pathways in T cells, we performed metabolomics profiling in Th9 cells from WT and *Hif1α*^*kd*^ mice to identify the key metabolites and metabolic pathways regulated by HIF1α essential for the generation of Th9 cells. To do this, we quantified metabolites of different metabolic pathways in cell extracts as well as cell-free culture supernatants (footprinting). Metabolomics data demonstrated a differential expression of metabolites in WT and *Hif1α*^*kd*^ Th9 cells (Fig. 7a). There was a decreased production of metabolites of glycolysis, pentose phosphate pathway (PPP), fatty acid pathway and energy metabolites in *Hif1α*^*kd*^ Th9 cells as compared to WT Th9 cells (Fig. 7a–c and Supplementary Fig. 8a–c). *Hif1α*^*kd*^ decreased ATP and lactate (Lac) production in Th9 cells (Fig. 7c, d). We further identified a striking decrease in α-ketoglutarate (αKG), a TCA cycle metabolite in *Hif1α*^*kd*^ Th9 cells (Fig. 7e) which led us to further investigate and validate its role in Th9 cell differentiation.

Metabolomics analysis illustrated a decrease in αKG production in *Hif1α*^*kd*^ Th9 cells, suggesting a possible link between the production of αKG and HIF1α in Th9 cells. It has been demonstrated that αKG increases PHD2 activity leading to inactivation of HIF1α in macrophages^50^. This led us to investigate the role of αKG in HIF1α-dependent Th9 cell differentiation. NanoString analysis of Th9 cells cultured in the presence of αKG showed the downregulation of key transcription factors and cytokines signature of Th9 cells (Fig. 7f). Consistently, we found that αKG inhibited the induction of both HIF1α and IL-9 in Th9 cells while led to an increase in *Egln1* expression (gene encoding for PHD2) which corresponds to an enhanced PHD2 activity (Fig. 7g–i).

αKG is subsequently converted to succinate through GABA transaminase (GABA-T)^50^. Succinate increases *Hif1α* expression and decreases PHD2 activity^50,51^. Vigabatrin, an irreversible inhibitor of GABA transaminase, leads to decreased succinate formation and increased accumulation of αKG eventually depleting HIF1α^50^. In line with these findings, we found that vigabatrin significantly decreased HIF1α and IL-9 induction possibly due to the accumulation of αKG in Th9 cells (Supplementary Fig. 9a, b).

Succinate is derived from glutamine either through anaplerosis via αKG or through “GABA shunt pathway”. Succinate is transported to the cytosol from the mitochondria where it creates ‘pseudohypoxia’ by impairing PHD2 activity leading to HIF1α stabilization and activation. This effect is blocked by αKG, the substrate for PHD2 that generates succinate as a by-product in HIF1α hydroxylation^52,53^. We have shown that αKG depletes HIF1α and suppresses IL-9 in Th9 cells. Next, we tested the effect of succinate on HIF1α and IL-9 induction, and found that succinate increases the expression of *Il9, Spi1, Irf4, Irf1, Gata3*, and IL-9 production in Th9 cells (Fig. 7j, k). We found that succinate impaired PHD2 activity by inhibiting *Egln1* expression, which resulted in increased *Hif1α* expression in Th9 cells, implying that succinate stabilizes HIF1α and enhances IL-9 induction in Th9 cells (Fig. 7l). There was no significant influence of αKG and succinate on the induction of IL-10 and IFN-γ respectively (Supplementary Fig. 10a–d). Moreover, in comparison to TGF-β1 or IL-4 alone, TGF-β1 + IL-4 together resulted in a greater increase in *Il9* expression in the presence of succinate in the Th9 cells (Supplementary Fig. 10e). Also, there was no differential *Il9* expression in Th0 cells in the presence or absence of succinate (Supplementary Fig. 10f). Finally, we examined the in vivo role of succinate in the anti-tumor functions of Th9 cells in B16-OVA melanoma tumor model. We found that the tumor volume was significantly decreased in mice which received OT-II-Th9 cells as compared to the B16-OVA control mice (Fig. 7m). Strikingly, in comparison to OT-II-Th9 cells, succinate-treated OT-II-Th9 cells led to a greater reduction in the tumor volume (Fig. 7m), implying that succinate enhances the differentiation and anti-tumor functions of Th9 cells through increased stabilization of HIF1α by impairing PHD2 activity.

**Fig. 8:**
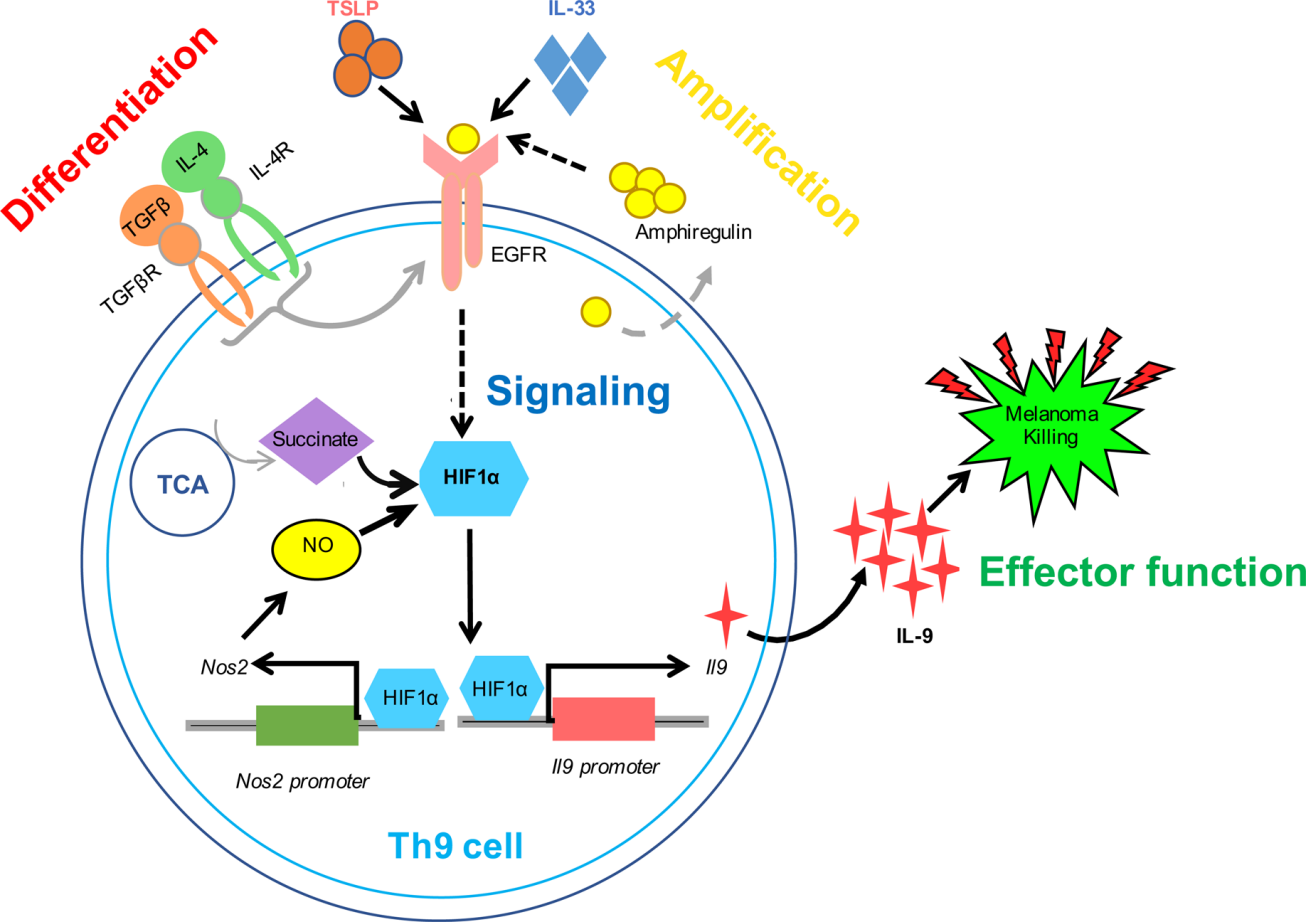
Schematic representation of EGFR-HIF1α signaling pathway in Th9 cells. TGF-β1 and IL-4 initiates the “differentiation” of naïve CD4^+^ T cells into Th9 cells, which expresses EGFR and produces EGFR ligand, Areg. Areg “amplifies” Th9 cell differentiation via activating EGFR in a feed-forward loop. Upon activation, EGFR triggers downstream “signaling” through HIF1α which transactivates *Il9* and *Nos2* promoters resulting in enhanced IL-9 induction. Succinate, a TCA cycle metabolite, and nitric oxide (NO) further stabilizes HIF1α potentiating Th9 cell differentiation and anti-tumor effector functions.

Based on the experimental data provided in this study, we propose a model for Th9 cell differentiation. TGF-β1 plus IL-4 initiates Th9 cell differentiation from naïve CD4^+^ T cells. Areg-EGFR signaling axis amplifies Th9 cell differentiation through EGFR-mediated activation of the transcription factor, HIF1α. HIF1α promotes Th9 cell differentiation: (i) metabolically through TCA cycle metabolite, Succinate, and (ii) transcriptionally by transactivating *Il9* and *Nos2* gene loci, further enhancing Th9 cell polarization and Th9 cell-mediated anti-tumor effector functions (Fig. 8).

## Discussion

The importance of Th9 cells in health and diseases is discussed for the reason that IL-9 and IL-9R are crucial for disease pathogenesis in allergic inflammation. It has been previously shown that genetic polymorphism in the *Il9* gene is linked with an increased risk of developing cutaneous malignant melanoma^54^. Subsequent studies clearly showed that Th9 cells mount more potent anti-tumor immunity as compared to Th1, Th2 and Th17 cells^22,27,28^. However, the generation of Th9 cells are still not completely understood which led to an impetus for unraveling the unknown molecular pathways in the development and functions of Th9 cells. Our RNA-Seq analysis identified a strong upregulation of EGFR pathway in mouse Th9 cells. Inhibition of EGFR suppressed the IL-9 induction in all Th cells and our data indicates that EGFR pathway is crucial for the anti-tumor functions of Th9 cells.

Activation of EGFR signaling is induced by phosphorylation, which subsequently activates downstream signaling components^30^. We found that Th9 cells produce Areg, an EGFR ligand, which has previously shown to play an important role in mediating effector and regulatory functions of Th2^31^ and Tregs^32^ respectively. Th9 shares gene program closer to Th2 and Tregs by virtue of the common differentiation factors such as TGF-β1 with Tregs and IL-4 with Th2 respectively. This suggests a possibility for the involvement of Areg in Th9 cells. Our data has identified that Areg enhances Th9 cell differentiation. EGFR-mediated IL-9 induction was significantly impaired in *Areg*^−/−^ mice in which the expression of *Egfr, Il9* and Th9-associated genes were suppressed. It is reported that EGFR activation leads to STAT3 activation, and STAT3 is a negative regulator of Th9 cells. Since the in vitro culture conditions for Th9 cell differentiation contains IL-4, it is possible that IL-4-induced STAT6 might antagonize the functions of STAT3 activated by Areg-EGFR, and as a result, Areg-EGFR-mediated activation of STAT3 may not be able to exert its negative effect on Th9 cell differentiation. In addition, Areg-EGFR signaling leads to the activation of NFκB, which is found to be essential for the differentiation of Th9 cells, as NFκB inhibition leads to the suppression of Th9 cells. It is, however, not clear whether Areg-EGFR-mediated activation of NFκB is dominant over STAT3 activation, and thus promotes Th9 cell differentiation.

Since IL-9 is also produced by other Th cells such as Th2, Th17, and iTregs^55^, we found that both Areg and EGFR are required for IL-9 induction in Th2, Th17, and iTregs as well. Thus, Areg-mediated EGFR signaling is required for IL-9 induction in all IL-9-producing Th cells. Further, we have shown that Th9-enhancing cytokines like IL-33 and TSLP enhances IL-9 induction in Th9 cells through increased expression of *Areg* and *Egfr*. This was evident when IL-33 could not completely rescue IL-9 induction in Th9 cells in the absence of EGFR. We also showed that NO is critical for *Egfr* expression and IL-9 induction in Th9 cells. Therefore, IL-33, TSLP, and NO serves as Th9-enhancing factors which functions through Areg-EGFR pathway.

Upon activation, EGFR signals through PI3K/AKT, RAS/MAPK pathways, which leads to cell proliferation, differentiation, and survival^30^. We further wanted to understand the downstream pathways of EGFR signaling that are important for triggering Th9 cell differentiation. Studies have shown a link between EGFR and HIF1α in tumor cells^34^, however, the role of EGFR-HIF1α axis has not been elucidated in T cells so far. Our NanoString analysis revealed a strong downregulation of the transcription factor, HIF1α in Th9 cells from *Egfr*^*flox/flox*^*XCd4-cre* mice as compared to WT Th9 cells. This compelled us to focus on HIF1α intriguingly for elucidating its role in the regulation of IL-9 induction in Th cells.

HIF1α and HIF2α are closely related isoforms of HIF, and both of these isoforms induce HRE-dependent gene expression^56^. Despite having similarities in their functions, knockout mice studies indicate non-redundant roles of HIF1α and HIF2α, and inactivation of one or other results in a distinctly different phenotype, which could be due to their tissue-specific and temporal expression^57^. Nonetheless, both of HIF1α and HIF2α isoforms could be also expressed in the same cells but may have different transcriptional targets. HIF1α, but not HIF2α, is the major factor that controls glycolytic pathway^58^. We and others have shown that Th9 cells expressed genes that are essential for glycolytic pathway^23,38^. In addition, it has been demonstrated that T cell activation primarily relies on the glycolytic pathway for fulfilling an increased energy demand and providing metabolic precursors required for cell survival and differentiation^49^. Though HIF1α is primarily essential for T cell activation and differentiation of effector T cells, emerging data indicates that HIF2α plays an indispensable role in regulatory T cell functions^59^.

Our RNA-Seq analysis demonstrated an upregulation of HIF1α, which is a critical transcription factor required for Th9 cell differentiation^23^. The glycolytic and hypoxia pathways were also found to be differentially expressed in Th9 cells. Corroborating with the published study, our findings also showed that HIF1α binds and transactivates *Il9* promoter resulting in enhanced Th9 cell differentiation. Physiologically, HIF1α inhibition repressed IL-9 induction in Th9 cells and subsequently promoted the tumor development in B16-OVA melanoma tumor model. In addition, we showed that HIF1α also binds to *Il9* promoter in other IL-9-producing Th cells such as Th2, Th17, and iTregs. IL-9 induction was significantly abrogated in Th9, Th2, Th17, and iTregs when HIF1α was knocked down suggesting that HIF1α globally regulates IL-9 induction in all IL-9 producing Th cells.

Prolyl hydroxylases 2 (PHD2) is known to degrade and destabilize HIF1α and so on knocking down *Phd2* gene, stability of HIF1α increases. We found that *Phd2* knockdown in Th9 cells resulted in increased expression of *Il9*, *Hif1α* and Th9-associated genes. *Phd2* knockdown also led to higher IL-9 induction in Th2, Th17, and iTregs. Consistent with our published human data, we also found a higher expression of *Hif1α, Il9*, and other signature genes in mouse Th9 cells under hypoxia as compared to normoxia. Furthermore, there was also an enhanced induction of IL-9 in Th2, Th17, and iTregs under hypoxia indicating that constitutive expression of HIF1α in hypoxic condition promotes enhanced IL-9 induction in all IL-9 producing Th cells.

Corroborating with the published literature, we found that there was an impaired induction of IL-9 and Th9-associated genes in *Nos2*^−/−^ Th9 cells. We also showed that *Nos2*^−/−^ abrogates IL-9 induction in Th2, Th17, and iTregs. Previously, we have shown that HIF1α and NO synergistically promote human Th9 cell differentiation^38^. Molecularly, we found that HIF1α binds and transactivates *Nos2* promoter in Th9 cells. Thus, there was a reduced *Nos2* expression in *Hif1α*^*kd*^ Th9 cells. Likewise, there was an increased expression of *Nos2* in Th9 cells differentiated under hypoxia or when *Phd2* was knocked down, both of which mimic constitutive expression of HIF1α in Th9 cells respectively. Furthermore, we found that NO, in turn, regulates HIF1α expression in Th9 cells since *Nos2*^−/−^ Th9 cells showed downregulated *Hif1α* expression. These data establish a functional interaction between HIF1α and NO in which both positively regulates each other potentiating Th9 cell differentiation synergistically. Moreover, we also found that EGFR-HIF1α pathway cooperatively regulates IL-9 induction in human Th9 cells. There was a higher induction of IL-9 and EGFR in human Th9 cells. EGFR inhibition substantially suppressed while Areg treatment significantly enhanced IL-9 induction in human Th9 cells.

HIF1α is a central metabolic regulator of T cell differentiation^49^, and we and others have shown that HIF1α is essential for glycolytic activity in both mouse and human Th9 cells^23,38^. Th9 cells are highly glycolytic as compared to other Th cells, however, the role of other metabolic pathways in Th9 cell differentiation remains unexplored. Thus, we sought to undertake a detailed understanding of different metabolic pathways in Th9 cells which are primarily regulated by HIF1α. Our whole cell metabolomics analysis of mouse Th9 cells from WT and *Hif1α*^*kd*^ mice identified decreased production of metabolites of TCA, PPP, fatty acid pathways, and energy metabolites apart from glycolysis in the absence of HIF1α. This reflects the importance of HIF1α as a key regulator of metabolic pathways in Th9 cells.

In addition, TCA cycle metabolite, α-Ketoglutarate (αKG), was also regulated by HIF1α in Th9 cells. It has been shown that αKG negatively regulates HIF1α stability through increased activation of *Egln1*, gene encoding for PHD2^50^. Here we showed that αKG increases the expression of *Egln1*, which decreases HIF1α activity resulting in the inhibition of Th9 cells. Furthermore, vigabatrin, which increases the accumulation of αKG, suppressed HIF1α and IL-9 induction in Th9 cells suggesting that αKG negatively regulates Th9 cell differentiation. Further, it has been shown that succinate promotes HIF1α stabilization by impairing PHD2 activity in macrophages^50^. Our data suggests a higher induction of IL-9, HIF1α, and Th9-associated genes in Th9 cells in the presence of succinate indicating that succinate positively regulates Th9 cell differentiation possibly by repressing PHD2 and enhancing HIF1α activity. Consequently, succinate treatment enhanced the anti-tumor potency of Th9 cells.

In summary, we have demonstrated the role of EGFR-HIF1α pathway in the differentiation of IL-9-producing Th cells. Th9 cells produce Areg, which activates EGFR resulting in the activation of the downstream HIF1α signaling pathway. NO and hypoxia stabilizes HIF1α which, in turn, induces NO potentiating Th9 cell differentiation. TCA cycle metabolite, Succinate promotes HIF1α stability and subsequently IL-9 induction in Th9 cells. Areg produced by Th9 cells further amplifies Th9 cell differentiation in a feed-forward loop. In conclusion, this study deciphers the molecular pathway involved in the regulation of IL-9 induction in Th cells and its subsequent implication in Th9 cell-mediated anti-tumor immune response, which could be potentially targeted for successful cancer immunotherapy.

## Methods

### Mice

C57BL/6 (#000664), OT-II TCR (#004194), and *Nos2*^−/−^ (#002596) mice were procured from Jackson laboratory, housed and maintained in a pathogen-free small animal facility (SAF) at the Translational Health Science and Technology Institute (THSTI), Faridabad, India. Mice were housed in individual ventilated cages supplemented with acidified water. The temperature for mice rooms at THSTI-SAF were maintained between ~19–26 °C with ~30–70% humidity. Mice were housed with 14 h light/10 h dark cycles. All mice experiments were performed in laminar floor hoods, and all personnel were required to wear personal protective equipment. *Egfr*^*flox/flox*^*XCd4-cre* mice were provided by D.M.W. Zaiss. The experiments on *Egfr*^*flox/flox*^*XCd4-cre* mice^32^ were performed at the University of Edinburgh in accordance with university ethical guidelines and the samples were shipped on dry ice to THSTI, India where subsequent assays were performed. *Areg*^−/−^ mice were provided by Fiona Powrie and *Phd2*^*kd*^ and *Hif1α*^*kd*^ mice were provided by Chris W. Pugh respectively. The experiments on *Areg*^*−/−*31^, *Phd2*^*kd*^ ^47^, and *Hif1α*^*kd*^ ^47^ were performed at Kennedy Institute of Rheumatology, University of Oxford, United Kingdom in accordance to the institutional ethical guidelines and the samples were shipped on dry ice to THSTI, India for performing further assays and analysis. All the mice used for experiments were 6–12 weeks old and both age and sex matched. All animal experiments were performed in accordance to the THSTI Animal Ethical guidelines.

### Transcriptome profiling using RNA sequencing

RNA extracted from the in vitro differentiated T cells was subjected to next-generation sequencing (NGS) to generate deep coverage RNA-Seq data. Sequencing libraries of Poly A selected mRNA were generated from the double-stranded cDNA using the IlluminaTruSeq kit according to the manufacturer’s protocol. Library quality control was checked using the Agilent DNA High Sensitivity Chip and qPCR. High quality libraries were sequenced on an Illumina HiSeq 2500. To achieve comprehensive coverage for each sample, ~25–30 million paired end reads were generated.

### RNA-Seq data analysis

The Quality check of the sequenced read were performed by FASTQC (version 0.11.9) and FASTX (version 0.0.13) to remove the adapter and unwanted low quality reads. Tophat2 and Bowtie2 packages were used to align the cleaned reads to the reference mouse genome (GRCm38). Subsequently, Htseq-count algorithm were used to measure gene expression from aligned reads. The read count-based gene expression data were normalized on the basis of library complexity and gene variation using the R package Cuffdiff. The normalized count data were compared between groups to identify differentially expressed genes. Genes were considered significantly differentially expressed if the *P*-value was >0.0001 FDR and absolute fold change cut-off was >2.^60,61^. The downstream analysis was done by in-house script (Supplementary data 1).

### Ingenuity Pathway analysis

Ingenuity Pathway Analysis (IPA 8.0, Qiagen) was used to identify the pathways that are significantly affected by differentially expressed genes. The knowledge base of this software consists of functions, pathways, and network models derived by systematically exploring the peer reviewed scientific literature. It calculates *P*-value for each pathway according to the fit of user’s data to the IPA database using one-tailed Fisher exact test. The pathways with multiple test corrected *P*-values <0.01 were considered significantly affected^62^.

### NanoString analysis

The NanoString experiments were performed as per the manufacture’s protocol. Briefly, ~80 ng of total RNA was isolated and hybridized with reporter and capture probes in custom-made T helper cell-targeted nCounter Gene Expression code set according to manufacturer’s instructions (NanoString Technologies). Data were analyzed using nSolver Analysis software^63^.

### In vitro mouse T helper cells differentiation

6–12 weeks old WT mice were euthanized and spleen and lymph nodes were collected aseptically. Single cell suspensions from spleen and lymph nodes were prepared after lysing red blood cells using ACK lysis buffer. Cells were then stained with the cell surface antibodies- anti-mouse CD4 PerCP (RM4-5; BioLegend Cat # 100538; 1:200), anti-mouse CD62L APC (MEL-14; BioLegend Cat # 104412, 1:200), and anti-mouse CD25 PE (3C7, BioLegend Cat # 101904, 1:200). Cells were sorted on BD FACS Aria III with approximately >98% purity.

Sorted purified naïve (CD4^+^CD62L^+^) T cells were activated with plate bound anti-CD3 (2.0 μg/ml; 145-2C11; Bio X Cell; Cat # BE0001-1) and anti-CD28 (2.0 μg/ml; 37.51; Bio X Cell; Cat # BE0015-1), and were in vitro differentiated using the following cytokines: Th1 [IL-12 (10 ng/ml)], Th2 [IL-4 (10 ng/ml)], Th9 [TGF-β1 (2.0 ng/ml), IL-4 (20 ng/ml)], Th17 [TGF-β1 (2.0 ng/ml), IL-6 (25 ng/ml)], and iTregs [TGF-β1 (2.0 ng/ml), IL-2 (50 U/ml)] for 3 days respectively. In addition, Areg (100 ng/ml), IL-33 (10 ng/ml) and TSLP (10 ng/ml) were added during differentiation, wherever indicated. Hypoxia experiments were carried out in a hypoxia chamber (Coy Laboratory Products) inside which cells were differentiated at 1.0% oxygen for 3 days.

### In vitro human T helper cells differentiation

10 ml of peripheral blood was collected from healthy human volunteers after written informed consent in accordance with the approval of the institutional human ethics committee. Peripheral blood mononuclear cells (PBMCs) were isolated from whole blood using ficoll-paque based density gradient centrifugation and were then stained with the following cell surface fluorochrome-labelled antibodies: anti-human CD4 Horizon V450 (RPA-T4; BD Biosciences Cat # 560345; 1:200), anti-human CD45RA PE/Cy7 (HI100; BioLegend Cat # 304126; 1:200) and anti-human CD45RO APC (UCHL1; BioLegend Cat # 304210; 1:200) and subjected to sorting on BD FACS Aria. Naïve CD4^+^ T cells (CD4^+^CD45RA^+^CD45RO^‒^) were sorted on BD FACS Aria III with >95% purity.

Naïve CD4^+^ T cells (CD4^+^CD45RA^+^CD45RO^−^) were activated with plate bound anti-hCD3 (10 μg/ml; OKT-3; Bio X Cell Cat # BE0001-2) and soluble anti-hCD28 (2.0 ug/ml; CD28.2; BD Biosciences Cat # 555725) for 6 days in the presence of TGF-β1 (2.0 ng/ml) and IL-4 (20 ng/ml) for Th9 differentiation. 100 ng/ml recombinant Areg was added during Th9 cell differentiation wherever indicated.

### qPCR

Differentiated T cells were lysed in RLT buffer (Qiagen), RNA was extracted using the RNAeasy Mini Kit (Qiagen; #74104) and reverse transcribed into cDNA using the iScript cDNA synthesis kit (Biorad; #1708891). qPCR was done using KAPA SYBR® FAST qPCR Master Mix (2X) Universal Kit (KK4600) on Fast 7500 Dx Real-Time PCR system (Applied Biosystems). Results were analyzed with SDS2.1 software. The cycling threshold value of the endogenous control genes, *β-actin* (for mouse) and *Gapdh* (for human) was subtracted from the cycling threshold value of each target gene to generate the change in cycling threshold (ΔCT). The relative expression of each target gene is expressed as “fold change” relative to that of unstimulated samples (2-ΔCT). We used the previously used formula (POWER(2,−ΔCT)*10,000 to calculate the relative gene expression^64^. The SYBR primers used for the analysis are mentioned in Supplementary Table 1.

### Cytokine ELISA

Cytokines were measured in the culture supernatants by sandwich enzyme-linked immunosorbent assay (ELISA) as described^64^. Plates were read at 405 nM and the absolute quantity of cytokines were determined using standard for the respective cytokines. ELISA for mouse Areg was performed using kit from Cloud-Clone (SEA006Mu).

### Intracellular cytokine staining and Flow cytometry

In vitro differentiated T cells were re-stimulated with PMA (phorbol 12-myristate13-acetate; 50 ng/ml; Sigma-Aldrich), ionomycin (1.0 μg/ml; Sigma-Aldrich), and monensin (GolgiStop, BD Biosciences Cat # 554724) for 6 h^64^. Cell surface staining was done for 15–20 min with anti-mouse CD4 [RM4-5; BioLegend (CD4 PerCP Cat # 100538, CD4 PE Cat #100512, CD4 APC Cat # 100516); 1:200] and anti-mouse CD8a PerCP (53-6.7; BioLegend Cat # 100732; 1:200) for mouse; and anti-human CD4 APC (OKT4, BioLegend Cat # 317416; 1:200) for human after live/dead marker staining respectively. For intracellular staining, cells were fixed in Cytofix solution and permeabilized with 1X Perm/Wash Buffer using kit (BD Biosciences Cat # 554714). Cells were then stained with anti-mouse IL-17A [TC11-18H10.1; BioLegend (IL-17A APC Cat # 506916, IL-17A PE/Cy7 Cat # 506922, IL-17A PerCP/Cy5.5 Cat # 506920, IL-17A Pacific Blue Cat # 506918, IL-17A FITC Cat # 506908; 1:200], anti-mouse IL-9 [RM9A4; BioLegend (IL-9 PE Cat # 514104, IL-9 PerCP/Cy5.5 Cat # 514112, IL-9 APC Cat # 514106) 1:200], anti-mouse IFN-γ PE/Cy7 (XMG1.2; BioLegend Cat # 505826; 1:200) or anti-human IL-9 [MH9A4; BioLegend (IL-9 PerCP/Cy5.5 Cat # 507610, IL-9 PE Cat # 507605) 1:200] in Perm/Wash buffer. The cells were acquired using flow cytometry on FACSCantoII with FACSDiva software version 8.0.2 (BD biosciences) or on FACSVerse (BD Biosciences) and the results were analyzed with FlowJo software version 10 (Tree Star).

### Luciferase reporter assay

HEK293T cells were transiently transfected with HA-HIF1α-pcDNA3 (Addgene; # 18949) and the respective promoters construct, pGL3-IL-9 promoter luciferase or pGL2-Nos2 promoter luciferase (Addgene; # 19296) tagged with firefly luciferase reporter, and renilla luciferase reporter vector using X-treme GENE^TM^ 9 DNA Transfection Reagent (# 06365787001; Roche). For pGL3-IL-9 promoter luciferase plasmid construct, the mouse *Il9* promoter region (NC_000079.6) from +7177 to +9277 was amplified from genomic DNA by PCR with the forward primer (5′-ATGCACGCGTTCTGTCAGAGAGAGGTGTAG-3′) and the reverse primer (5′-ATG CCCCGGGTCAGTCTACCAGCATCTTCC-3′). The amplified fragment was cloned into the pGL3 basic luciferase reporter gene vector (Promega) at MluI and SmaI restriction sites. Luciferase luminescence was measured after 48 h of transfection by using Dual Glo Luciferase Reporter Assay system as per manufacturer’s protocol (Promega; E2940). Firefly luciferase activity was normalized to renilla luciferase activity and the result was represented as relative light units (RLU).

### Chromatin immunoprecipitation (ChIP)

1 × 10^7^ naïve mouse CD4^+^ T cells were differentiated in vitro into Th2, Th9, Th17, and iTregs for 3 days followed by cross-linking with 1% formaldehyde to preserve the DNA-protein interactions. Samples were further processed for enzymatic digestion of chromatin using SimpleChIP^®^ Enzymatic Chromatin IP Kit (Cell signaling technology; Cat # 9003S). Lysates were sonicated and immunoprecipitated with anti-HIF1α antibody (Abcam; Cat # ab228649; 5.0 μg per immunoprecipitation) or Rabbit IgG Isotype Control (ChIP grade) antibody (Abcam; Cat # ab171870; 1.0 μg per immunoprecipitation) respectively. DNA was eluted after reverse cross-linking of immunoprecipitated complex followed by qPCR. The putative binding sites of HIF1α on IL-9P and Nos2P promoters were amplified respectively by quantitative qPCR (SYBR Green chemistry). The values were subtracted from the amount of isotype IgG negative control and were normalized to the corresponding input control. The results were expressed as percent of total input control. List of the primers used for amplification are mentioned in Supplementary Table 2.

### Mass spectrometry-based metabolomics analysis using ion chromatography

Naïve CD4^+^ T cells from WT and *Hif1α*^*kd*^ mice were differentiated in vitro into Th9 cells for 3 days. 1 × 10^6^ cells were incubated in RPMI (glucose-free formulation) containing 10 mM [U-^13^C] glucose (Cambridge Isotope Laboratories), 2.0 mM glutamine, and 10% dialyzed FBS at 37 °C for 1 h. Cells were washed in 150 mM of ice-cold ammonium acetate (pH 7.3) and metabolites were extracted in 80% methanol on dry ice followed by evaporation under vacuum. Dried metabolites were resuspended in 50% acrylonitrile (ACN) and 1/10th was loaded onto a Luna 3 μm NH2 100 A (150 × 2.0 mm) column (Phenomenex). The chromatographic separation was performed using an UltiMate 3000 RSLC (Thermo Scientific) with mobile phases A (5 mM NH4AcO pH 9.9) and B (ACN) and a flow rate of 200 μl/min. The gradient from 15% A to 95% A over 18 min was followed by 9 min isocratic flow at 95% A and re-equilibration to 15% A. Metabolite detection was achieved with a Thermo Scientific Q Exactive mass spectrometer run with polarity switching in Full Scan mode with an m/z range of 65–975. TraceFinder 4.1 (Thermo Scientific) was used to quantify metabolites by area under the curve using retention time and accurate mass measurements (<3 ppm). Moreover, cell-free culture supernatants were also collected and subjected to mass spectrometry-based metabolomics analysis using ion chromatography for the quantification of secreted metabolites (metabolic footprinting). Data analysis was performed using in-house scripts in the statistical language R. Statistical differences were determined by one-way ANOVA testing.

### Adoptive transfer and B16-OVA melanoma tumor model

B16F10 melanoma cell line expressing ovalbumin (B16-OVA) was grown in RPMI 1640 supplemented with 10% FBS and 100 U/ml Pen/Strep. 2 × 10^5^ B16-OVA cells were subcutaneously injected into the right flank region of 6–8-week-old female WT mice for the development of melanoma. To investigate the in vivo role of EGFR, HIF1α and succinate in Th9 cell-mediated anti-tumor immune response, naïve CD4^+^ T cells from OT-II TCR transgenic mice (which specifically recognize OVA) were differentiated into Th9 cells with or without gefitinib, acriflavine, and succinate respectively, added at the indicated concentrations. 2 × 10^6^ OVA-specific Th9 cells (±gefitinib, acriflavine, succinate) were intravenously injected into the B16-OVA tumor-bearing mice respectively at day 5 after the appearance of small palpable tumor.

Tumor growth was monitored and tumor volume was measured every 2 days using vernier caliper. Tumor volume was calculated as: volume (mm^3^) = *L* × *W*^2^ /2, where *L* is the length and *W* is the width of the tumor (in mm). Mice were euthanized when the tumor volume exceeded 2000 mm^3^ or there was severe skin necrosis defined as the end point of the study^22,28^. Spleen (Spl) and tumor draining lymph nodes (dLN) were aseptically removed from all the mice at the end point and were further processed to make single cell suspension. Cells were re-stimulated ex vivo with PMA and ionomycin for intracellular cytokine staining for analyzing the expression of CD4^+^ and CD8^+^ T cells producing IFN-γ using flow cytometry as described^22,28^.

### Isolation of tumor-infiltrating lymphocytes (TILs)

In adoptive transfer B16-OVA melanoma tumor model experiment, mice were euthanized and tumors were excised, enzymatically digested with collagenase D (Roche; 11088858001) followed by mechanical disruption using gentleMACS^TM^ Dissociator (Miltenyi Biotec)^28^. The cells were washed with media and passed through 40 µm strainer and subjected to percoll density gradient centrifugation yielding separate layers out of which a faint layer near to 63% percoll gradient in the tube was collected and washed twice with 1x PBS. The resulting cell pellet consists of TILs^28^. TILs were further re-stimulated ex vivo with PMA/ionomycin for six hours and stained with respective fluorochrome-tagged FACS antibodies for intracellular cytokine staining for analyzing the expression of CD4^+^ and CD8^+^ T cells producing IFN-γ using flow cytometry as described^28^.

### Statistical analysis

GraphPad Prism 7.0 software was used for statistical analysis. Unpaired two-tailed Student’s *t* test for comparison of means between two groups; one-way ANOVA for comparison of means between more than two groups and two-way ANOVA test for comparison among multiple groups with two variables were used. All the statistical tests were followed by Tukey’s multiple comparison’s post-test. Differences were considered statistically significant with a *P*-value < 0.05 for all the experiments. All the data depicted in the bar graphs and scatter dot plots are represented as mean ± SEM.

### Reporting summary

Further information on research design is available in the Nature Research Reporting Summary linked to this article.

## Supplementary information

Supplementary Information

Description of Additional Supplementary Files

Supplementary Data 1

Reporting summary

## Data Availability

Sequence data that support the findings of this study have been deposited in GEO with the primary accession code, GSE163056. Publicly available data with accession code, GSE100634, were reanalyzed. The authors declare that all other data supporting the findings of this study are available within the article and its supplementary information files.

## References

[1] Dardalhon V (2008). IL-4 inhibits TGF-beta-induced Foxp3+ T cells and, together with TGF-beta, generates IL-9+ IL-10+ Foxp3(-) effector T cells. Nat. Immunol..

[2] Veldhoen M (2008). Transforming growth factor-beta ‘reprograms’ the differentiation of T helper 2 cells and promotes an interleukin 9-producing subset. Nat. Immunol..

[3] Schmitt E (1994). IL-9 production of naive CD4+ T cells depends on IL-2, is synergistically enhanced by a combination of TGF-beta and IL-4, and is inhibited by IFN-gamma. J. Immunol..

[4] Froidure A (2014). Myeloid dendritic cells are primed in allergic asthma for thymic stromal lymphopoietin-mediated induction of Th2 and Th9 responses. Allergy.

[5] Liu JQ (2015). Tumor-specific Th2 responses inhibit growth of CT26 colon-cancer cells in mice via converting intratumor regulatory T cells to Th9 cells. Sci. Rep..

[6] Maier E, Werner D, Duschl A, Bohle B, Horejs-Hoeck J (2014). Human Th2 but not Th9 cells release IL-31 in a STAT6/NF-kappaB-dependent way. J. Immunol..

[7] Schmitt E (1991). IL-1 serves as a secondary signal for IL-9 expression. J. Immunol..

[8] Xue G, Jin G, Fang J, Lu Y (2019). IL-4 together with IL-1beta induces antitumor Th9 cell differentiation in the absence of TGF-beta signaling. Nat. Commun..

[9] Angkasekwinai P, Chang SH, Thapa M, Watarai H, Dong C (2010). Regulation of IL-9 expression by IL-25 signaling. Nat. Immunol..

[10] Blom L, Poulsen BC, Jensen BM, Hansen A, Poulsen LK (2011). IL-33 induces IL-9 production in human CD4+ T cells and basophils. PLoS ONE.

[11] Yao W (2013). Interleukin-9 is required for allergic airway inflammation mediated by the cytokine TSLP. Immunity.

[12] Gessner A, Blum H, Rollinghoff M (1993). Differential regulation of IL-9-expression after infection with Leishmania major in susceptible and resistant mice. Immunobiology.

[13] Beriou G (2010). TGF-beta induces IL-9 production from human Th17 cells. J. Immunol..

[14] Elyaman W (2009). IL-9 induces differentiation of TH17 cells and enhances function of FoxP3+ natural regulatory T cells. Proc. Natl Acad. Sci. USA.

[15] Nowak EC (2009). IL-9 as a mediator of Th17-driven inflammatory disease. J. Exp. Med..

[16] Lu LF (2006). Mast cells are essential intermediaries in regulatory T-cell tolerance. Nature.

[17] Goswami R, Kaplan MH (2011). A brief history of IL-9. J. Immunol..

[18] Chang HC (2010). The transcription factor PU.1 is required for the development of IL-9-producing T cells and allergic inflammation. Nat. Immunol..

[19] Staudt V (2010). Interferon-regulatory factor 4 is essential for the developmental program of T helper 9 cells. Immunity.

[20] Jabeen R (2013). Th9 cell development requires a BATF-regulated transcriptional network. The. J. Clin. Investig..

[21] Goswami R (2012). STAT6-dependent regulation of Th9 development. J. Immunol..

[22] Vegran F (2014). The transcription factor IRF1 dictates the IL-21-dependent anticancer functions of TH9 cells. Nat. Immunol..

[23] Wang Y (2016). Histone deacetylase SIRT1 negatively regulates the differentiation of interleukin-9-producing CD4(+) T cells. Immunity.

[24] Wilhelm C (2011). An IL-9 fate reporter demonstrates the induction of an innate IL-9 response in lung inflammation. Nat. Immunol..

[25] Nalleweg N (2015). IL-9 and its receptor are predominantly involved in the pathogenesis of UC. Gut.

[26] Licona-Limon P (2013). Th9 cells drive host immunity against gastrointestinal worm infection. Immunity.

[27] Lu Y (2012). Th9 cells promote antitumor immune responses in vivo. J. Clin. Investig..

[28] Purwar R (2012). Robust tumor immunity to melanoma mediated by interleukin-9-producing T cells. Nat. Med..

[29] Eyles J (2010). Tumor cells disseminate early, but immunosurveillance limits metastatic outgrowth, in a mouse model of melanoma. J. Clin. Investig..

[30] Yarden Y, Sliwkowski MX (2001). Untangling the ErbB signalling network. Nat. Rev. Mol. Cell Biol..

[31] Zaiss DM (2006). Amphiregulin, a TH2 cytokine enhancing resistance to nematodes. Science.

[32] Zaiss DM (2013). Amphiregulin enhances regulatory T cell-suppressive function via the epidermal growth factor receptor. Immunity.

[33] Minutti CM (2017). Epidermal growth factor receptor expression licenses type-2 helper T cells to function in a T cell receptor-independent fashion. Immunity.

[34] Wang G (2018). ROS mediated EGFR/MEK/ERK/HIF-1alpha Loop Regulates Glucose metabolism in pancreatic cancer. Biochem. Biophys. Res. Commun..

[35] McNamee EN, Korns Johnson D, Homann D, Clambey ET (2013). Hypoxia and hypoxia-inducible factors as regulators of T cell development, differentiation, and function. Immunol. Res.

[36] Shi LZ (2011). HIF1alpha-dependent glycolytic pathway orchestrates a metabolic checkpoint for the differentiation of TH17 and Treg cells. J. Exp. Med..

[37] Dang EV (2011). Control of T(H)17/T(reg) balance by hypoxia-inducible factor 1. Cell.

[38] Roy S, Awasthi A (2019). ATP triggers human Th9 cell differentiation via nitric oxide-mediated mTOR-HIF1alpha pathway. Front. Immunol..

[39] Gordan JD, Thompson CB, Simon MC (2007). HIF and c-Myc: sibling rivals for control of cancer cell metabolism and proliferation. Cancer Cell.

[40] Ramadan A (2017). Specifically differentiated T cell subset promotes tumor immunity over fatal immunity. J. Exp. Med..

[41] Niedbala W (2014). Nitric oxide enhances Th9 cell differentiation and airway inflammation. Nat. Commun..

[42] Lee K (2009). Acriflavine inhibits HIF-1 dimerization, tumor growth, and vascularization. Proc. Natl Acad. Sci. USA.

[43] Di Conza G (2017). The mTOR and PP2A pathways regulate PHD2 phosphorylation to fine-tune HIF1alpha levels and colorectal cancer cell survival under hypoxia. Cell Rep..

[44] Ivan M (2001). HIFalpha targeted for VHL-mediated destruction by proline hydroxylation: implications for O_2_ sensing. Science.

[45] Yu F, White SB, Zhao Q, Lee FS (2001). HIF-1alpha binding to VHL is regulated by stimulus-sensitive proline hydroxylation. Proc. Natl Acad. Sci. USA.

[46] Jaakkola P (2001). Targeting of HIF-alpha to the von Hippel-Lindau ubiquitylation complex by O_2_-regulated prolyl hydroxylation. Science.

[47] Yamamoto A (2019). Systemic silencing of PHD2 causes reversible immune regulatory dysfunction. J. Clin. Investig..

[48] Hagen T, Taylor CT, Lam F, Moncada S (2003). Redistribution of intracellular oxygen in hypoxia by nitric oxide: effect on HIF1alpha. Science.

[49] Roy S, Rizvi ZA, Awasthi A (2018). Metabolic checkpoints in differentiation of helper T cells in tissue inflammation. Front. Immunol..

[50] Tannahill GM (2013). Succinate is an inflammatory signal that induces IL-1beta through HIF-1alpha. Nature.

[51] Qutub AA, Popel AS (2008). Reactive oxygen species regulate hypoxia-inducible factor 1alpha differentially in cancer and ischemia. Mol. Cell. Biol..

[52] Selak MA (2005). Succinate links TCA cycle dysfunction to oncogenesis by inhibiting HIF-alpha prolyl hydroxylase. Cancer Cell.

[53] Koivunen P (2007). Inhibition of hypoxia-inducible factor (HIF) hydroxylases by citric acid cycle intermediates: possible links between cell metabolism and stabilization of HIF. J. Biol. Chem..

[54] Yang XR (2009). Identification of modifier genes for cutaneous malignant melanoma in melanoma-prone families with and without CDKN2A mutations. Int. J. Cancer.

[55] Kaplan MH, Hufford MM, Olson MR (2015). The development and in vivo function of T helper 9 cells. Nat. Rev. Immunol..

[56] Wenger RH (2002). Cellular adaptation to hypoxia: O_2_-sensing protein hydroxylases, hypoxia-inducible transcription factors, and O_2_-regulated gene expression. FASEB J..

[57] Rosenberger C (2002). Expression of hypoxia-inducible factor-1alpha and -2alpha in hypoxic and ischemic rat kidneys. J. Am. Soc. Nephrol..

[58] Hu CJ, Wang LY, Chodosh LA, Keith B, Simon MC (2003). Differential roles of hypoxia-inducible factor 1alpha (HIF-1alpha) and HIF-2alpha in hypoxic gene regulation. Mol. Cell. Biol..

[59] Hsu TS (2020). HIF-2alpha is indispensable for regulatory T cell function. Nat. Commun..

[60] Raof NA (2016). The effects of transfection reagent polyethyleneimine (PEI) and non-targeting control siRNAs on global gene expression in human aortic smooth muscle cells. BMC Genomics.

[61] Tran MT (2016). PGC1alpha drives NAD biosynthesis linking oxidative metabolism to renal protection. Nature.

[62] Yosef N (2013). Dynamic regulatory network controlling TH17 cell differentiation. Nature.

[63] Rothhammer V (2018). Microglial control of astrocytes in response to microbial metabolites. Nature.

[64] Lee Y (2012). Induction and molecular signature of pathogenic TH17 cells. Nat. Immunol..

