# Supplementary material for: EGFR-HIF1α signaling positively regulates the differentiation of IL-9 producing T helper cells
Source: Nat Commun. 2021 Jun 1;12:3182. doi: 10.1038/s41467-021-23042-x (PMC8169867; doi:10.1038/s41467-021-23042-x)
Supplement: Supplementary file 1 — Supplementary Information [file 41467_2021_23042_MOESM1_ESM.pdf]

EGFR-HIF1α signaling positively regulates the differentiation of IL-9 producing T helper cells

Suyasha Roy, Zaigham Abbas Rizvi, Alexander J. Clarke, Felicity Macdonald, Abhaydeep Pandey, Dietmar Martin Werner Zaiss, Anna Kathrina Simon, Amit Awasthi

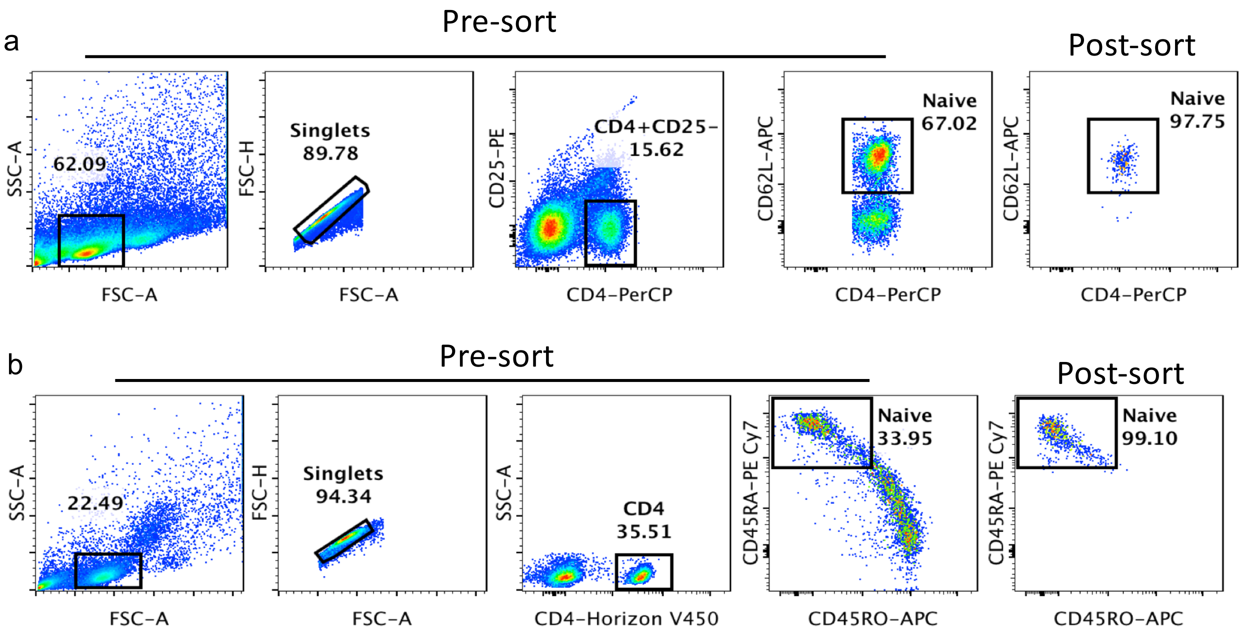

Supplementary Fig. 1. Gating and sorting strategy of mouse and human naïve CD4$^+$ T cells.

a. Spleen and lymph nodes were harvested from mice and single cell suspension were prepared. In the first step, lymphocytes were gated in forward and side scatter (SSC-A vs. FSC-A), and lymphocytes were further selected as singlets using the size discrimination parameters (FSC-H vs. FSC-A). Using the CD4, CD25 and CD62L fluorochrome labelled antibodies, CD25$^+$ and CD4$^-$ populations were excluded resulting in the selection of CD4$^+$ T cells, which was further sorted into CD4$^+$CD62L$^+$ (naïve T cells) cells while excluding the CD4$^+$CD62L$^-$ population and the purity of post-sort population, CD4$^+$CD62L$^+$ T cells, was determined as indicated. b. Blood from healthy individuals were collected and PBMCs were subjected to lymphocytes gating based on the forward and side scatter (SSC-A vs. FSC-A). Cells were further selected as singlets using the size discrimination parameter (FSC-H vs. FSC-A). Using the fluorochrome labelled anti-human CD4, CD45RA and CD45RO antibodies, naive CD4$^+$CD45RA$^+$ T cells were sorted with a 99% post-sort purity.

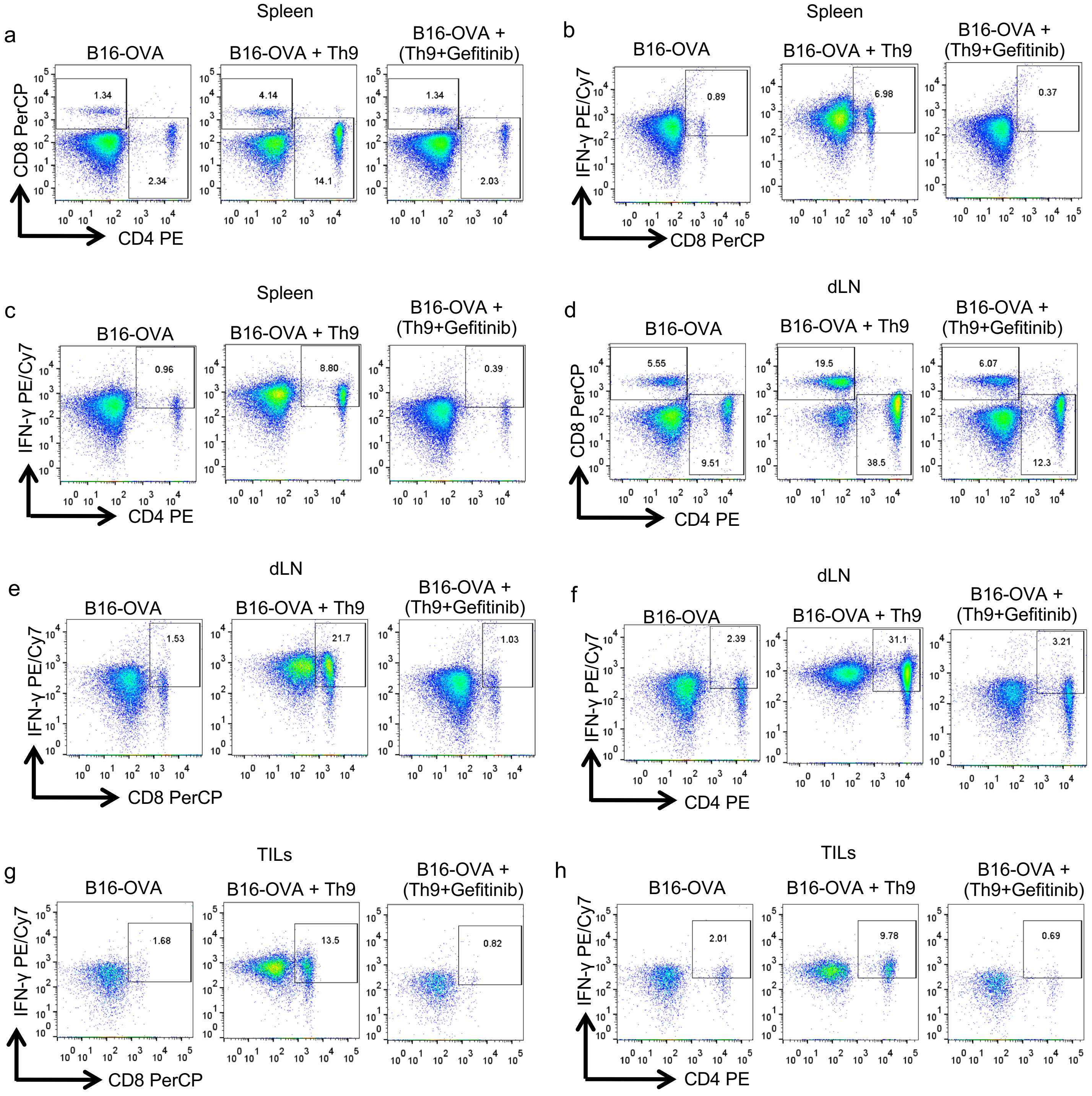

Supplementary Fig. 2. EGFR inhibition attenuates Th9 cell-mediated anti-tumor response *in vivo*.

a-h. Naïve $CD4^+$ T cells from OT-II TCR transgenic mice were *in vitro* differentiated into Th9 with or without 1.0 μM gefitinib for 3 days. Cells were then adoptively transferred into B16-OVA tumor bearing WT mice, randomized into three groups (n=5 mice per group). a-f. Representative FACS analysis for $CD4^+$, $CD8^+$, IFN-γ^+ populations in spleen and tumor draining lymph nodes (dLN). g,h. Tumor-infiltrating lymphocytes (TILs) were isolated from the tumor followed by FACS analysis of intracellular staining for $CD8^+$IFN-γ^+ and $CD4^+$IFN-γ^+ cell populations. Data are representative from three independent experiments.

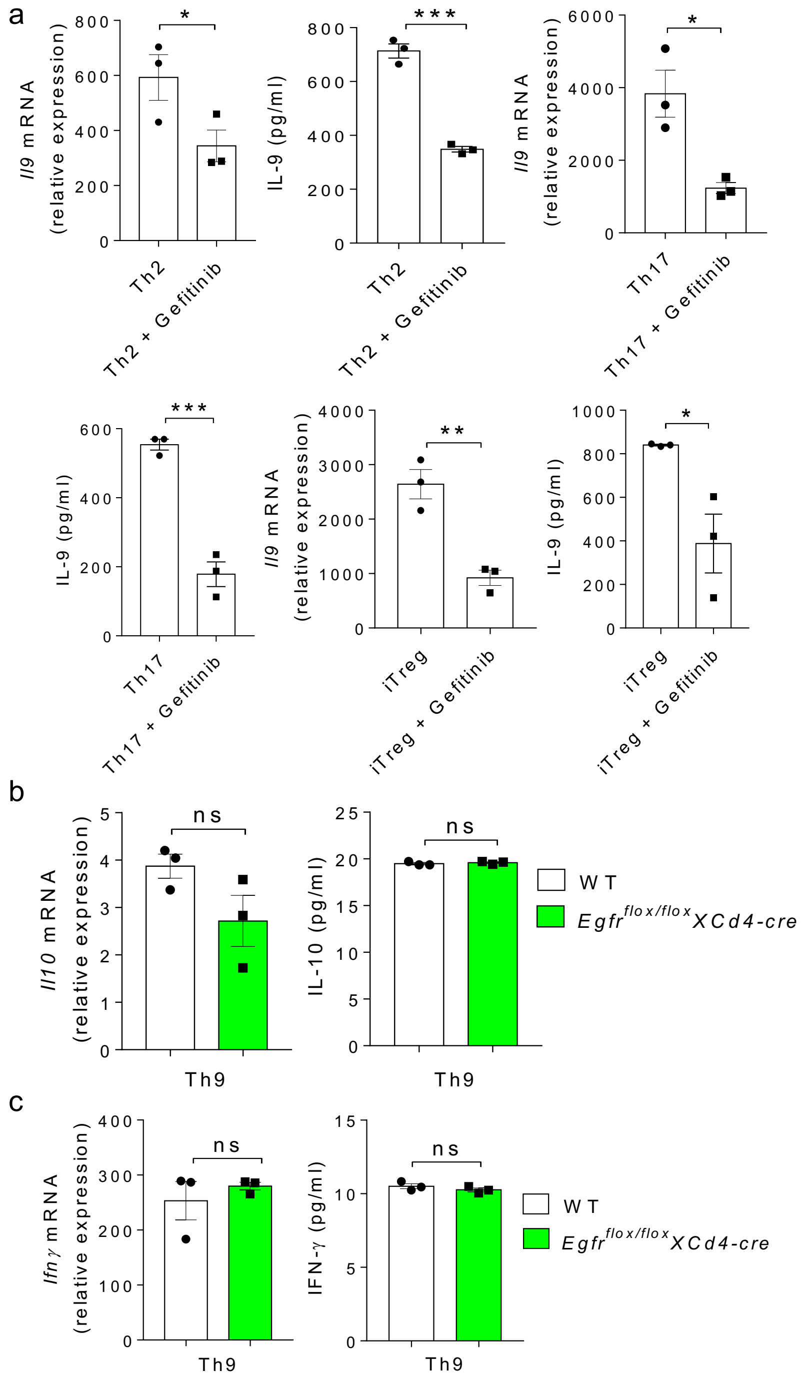

Supplementary Fig. 3. Blocking EGFR signaling abrogates IL-9 induction in Th2, Th17, iTregs.

a. Naïve $CD4^+$ T cells from WT mice were *in vitro* differentiated under Th2, Th17 and iTreg polarizing conditions with or without 1.0 µM gefitinib; analysis of IL-9 expression was done by qPCR and ELISA. Data are representative of mean ± SEM from three independent experiments. b,c. Naïve $CD4^+$ T cells from WT and *Egfr*$^{flox/flox}$*XCd4-cre* mice were differentiated under Th9 polarizing conditions for 3 days followed by qPCR and ELISA for IL-10 and IFN-γ. Data are representative of mean ± SEM from three independent experiments. a-c. *P < 0.0332, **P < 0.0021, ***P < 0.0002, *P* = ns (not significant), using two-tailed unpaired student's t test.

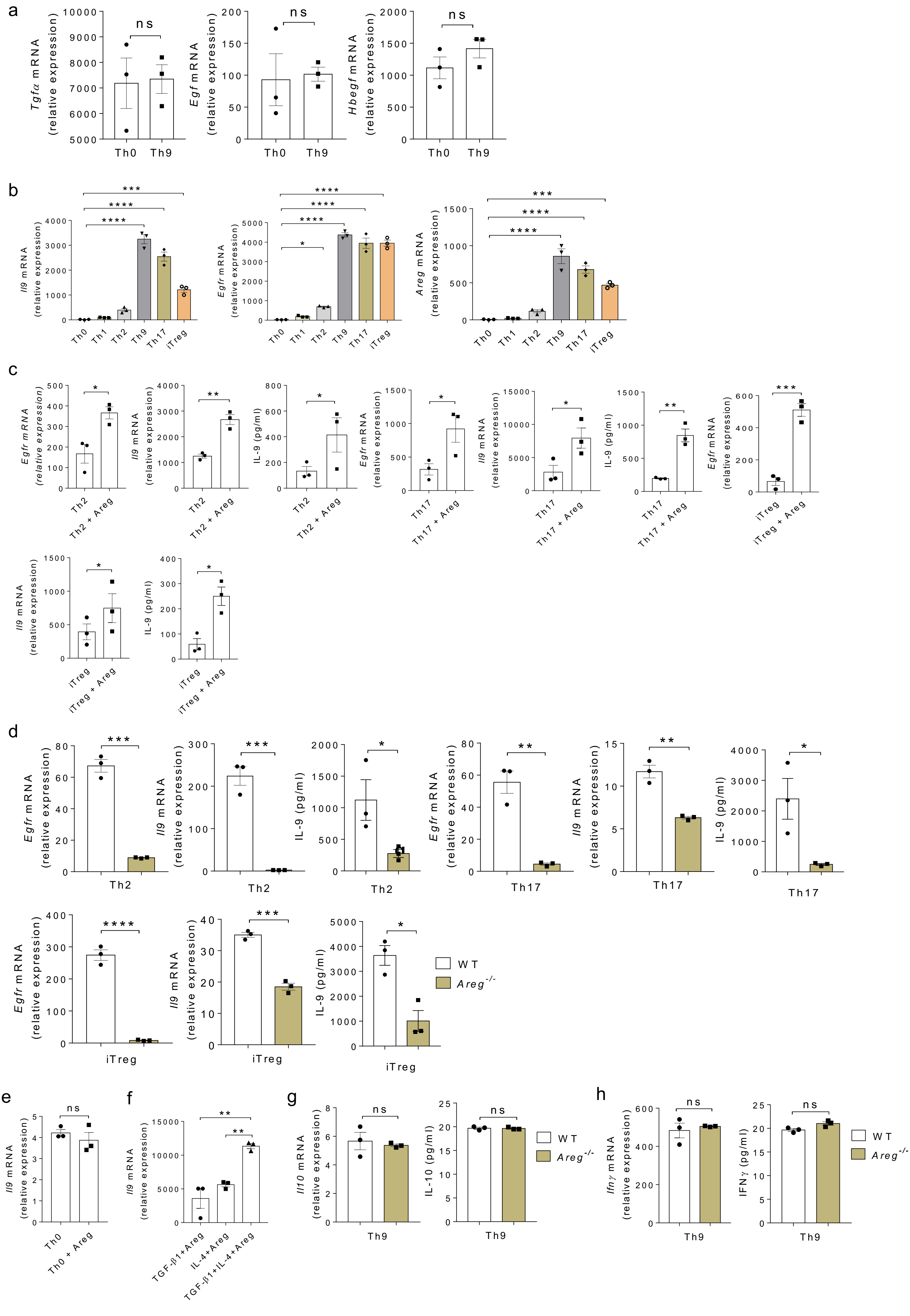
a
Tgfα mRNA (relative expression)
Egf mRNA (relative expression)
Hbegf mRNA (relative expression)
ns
Th0
Th9
b
Il9 mRNA (relative expression)
Egfr mRNA (relative expression)
Areg mRNA (relative expression)
Th0
Th1
Th2
Th9
Th17
iTreg
c
Egfr mRNA (relative expression)
Il9 mRNA (relative expression)
IL-9 (pg/ml)
Th2
Th2 + Areg
Th17
Th17 + Areg
iTreg
iTreg + Areg
d
Egfr mRNA (relative expression)
Il9 mRNA (relative expression)
IL-9 (pg/ml)
Th2
Th17
iTreg
WT
Areg-/-
e
Il9 mRNA (relative expression)
ns
Th0
Th0 + Areg
f
Il9 mRNA (relative expression)
TGF-β1+Areg
IL-4+Areg
TGF-β1+IL-4+Areg
g
Il10 mRNA (relative expression)
IL-10 (pg/ml)
Th9
WT
Areg-/-
h
Ifnγ mRNA (relative expression)
IFNγ (pg/ml)
Th9
WT
Areg-/-

Supplementary Fig. 4. Areg amplifies IL-9 induction in Th2, Th17, iTregs.

a. Naïve $CD4^+$ T cells from WT mice were differentiated into Th0 and Th9 followed by qPCR analysis of *Tgfα, Egf* and *Hbegf* expression. Data are representative of mean ± SEM from three independent experiments. b. Naïve $CD4^+$ T cells from WT mice were differentiated into Th0, Th1, Th2, Th9, Th17, iTregs followed by qPCR analysis of *Il9, Egfr* and *Areg* expression. Data are representative of mean ± SEM from three independent experiments c. Naïve $CD4^+$ T cells from WT mice were *in vitro* differentiated under Th2, Th17 and iTreg polarizing conditions with or without 100 ng/ml Areg followed by qPCR analysis of *Egfr* and *Il9* expression and ELISA for IL-9. Data are representative of mean ± SEM from three independent experiments. d. Naïve $CD4^+$ T cells from WT and $Areg^{-/-}$ mice were differentiated under Th2, Th17, iTreg polarizing conditions for 3 days followed by qPCR analysis of *Il9* and *Egfr* expression and ELISA for IL-9. Data are representative of mean ± SEM from three independent experiments e. Naïve $CD4^+$ T cells from WT mice were cultured as Th0 in the presence or absence of 100 ng/ml Areg followed by qPCR analysis of *Il9* expression. Data are representative of mean ± SEM from three independent experiments. f. Naïve $CD4^+$ T cells from WT mice were differentiated in the presence of TGF-β1 or IL-4 or TGF-β1+IL-4 with 100 ng/ml Areg supplementation followed by qPCR analysis of *Il9* expression. Data are representative of mean ± SEM from three independent experiments. g,h. Naïve $CD4^+$ T cells from WT and $Areg^{-/-}$ mice were differentiated under Th9 polarizing conditions for 3 days followed by qPCR and ELISA for IL-10 and IFN-γ. Data are representative of mean ± SEM from three independent experiments. a,c,d,e,g,h. *P < 0.0332, **P < 0.0021, ***P < 0.0002, ****P < 0.0001 , P = ns (not significant), using two-tailed unpaired student's t test. b,f. *P < 0.0332, **P < 0.0021, ***P < 0.0002, ****P < 0.0001, using one-way ANOVA followed by Tukey's multiple comparison test.

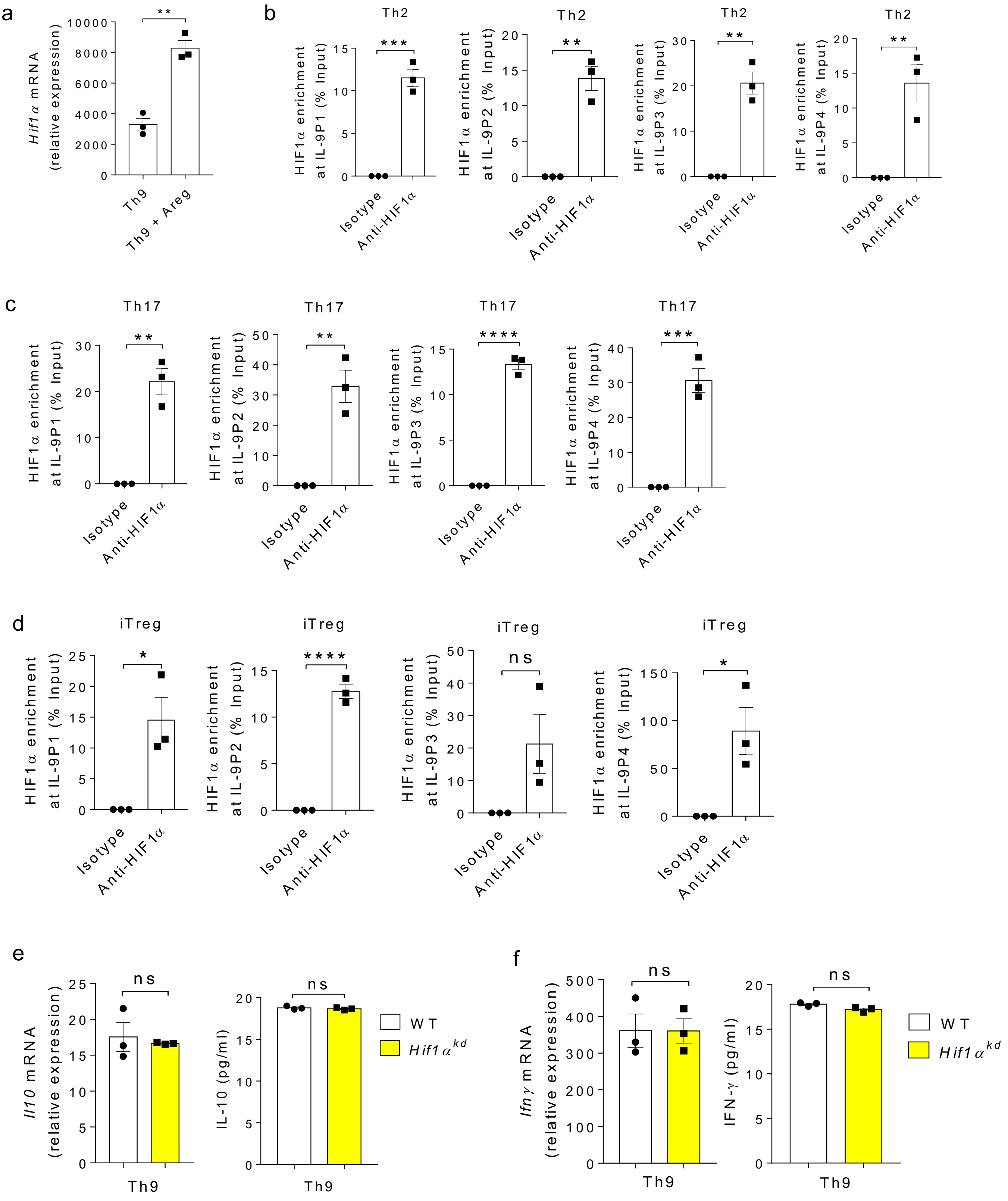

a
Hif1α mRNA (relative expression)
10000
8000
6000
4000
2000
0
**
Th9
Th9 + Areg
b
Th2
HIF1α enrichment at IL-9P1 (% Input)
HIF1α enrichment at IL-9P2 (% Input)
HIF1α enrichment at IL-9P3 (% Input)
HIF1α enrichment at IL-9P4 (% Input)
***
**
Isotype
Anti-HIF1α
c
Th17
HIF1α enrichment at IL-9P1 (% Input)
HIF1α enrichment at IL-9P2 (% Input)
HIF1α enrichment at IL-9P3 (% Input)
HIF1α enrichment at IL-9P4 (% Input)
**
****
***
Isotype
Anti-HIF1α
d
iTreg
HIF1α enrichment at IL-9P1 (% Input)
HIF1α enrichment at IL-9P2 (% Input)
HIF1α enrichment at IL-9P3 (% Input)
HIF1α enrichment at IL-9P4 (% Input)
*
****
ns
Isotype
Anti-HIF1α
e
Il10 mRNA (relative expression)
IL-10 (pg/ml)
ns
Th9
WT
Hif1α kd
f
Ifnγ mRNA (relative expression)
IFN-γ (pg/ml)
ns
Th9
WT
Hif1α kd

Supplementary Fig. 5. HIF1α binds to IL-9 promoter in Th2, Th17, iTregs.

a. Naïve $CD4^+$ T cells from WT mice were differentiated to Th9 in the presence or absence of 100 ng/ml Areg followed by qPCR analysis of *Hif1α* expression. Data are representative of mean ± SEM from three independent experiments. b-d. ChIP analysis of HIF1α binding to IL-9 promoter in Th2, Th17, iTregs represented as enrichment of HIF1α on IL-9 promoter relative to input. Data are representative of mean ± SEM from three independent experiments. e,f. Naïve $CD4^+$ T cells from WT and $Hif1\alpha^{kd}$ mice were differentiated under Th9 polarizing conditions for 3 days followed by qPCR and ELISA for IL-10 and IFN-γ. Data are representative of mean ± SEM from three independent experiments. a-f. *P < 0.0332, **P < 0.0021, ***P < 0.0002, ****P < 0.0001, *P* = ns (not significant), using two-tailed unpaired student's t test.

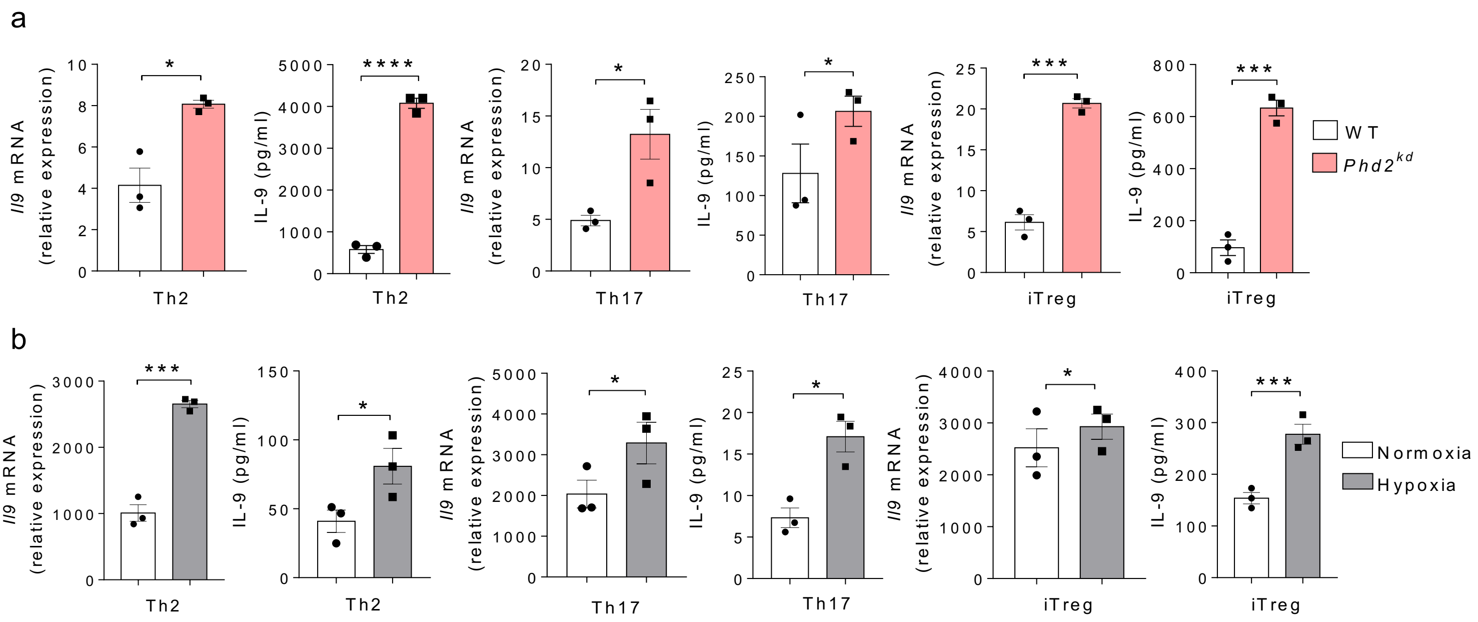

Supplementary Fig. 6. PHD2 and Hypoxia enhances IL-9 induction in Th2, Th17, iTregs.

a. Naïve CD4^{+} T cells from WT and *Phd2^{kd}* mice were differentiated under Th2, Th17, iTreg polarizing conditions with daily treatment of 1.0 µg/ml Dox for 3 days followed by qPCR and ELISA for IL-9. Data are representative of mean $\pm$ SEM from three independent experiments.

b. Naïve CD4^{+} T cells from WT mice were differentiated into Th2, Th17, iTregs under normoxic (21% oxygen) or hypoxic (1% oxygen) conditions for 3 days. qPCR analysis of *Il9* expression and ELISA for IL-9 production. Data are representative of mean $\pm$ SEM from three independent experiments. a.b. *P < 0.0332, ***P < 0.0002, ****P < 0.0001, using two-tailed unpaired student's t test.

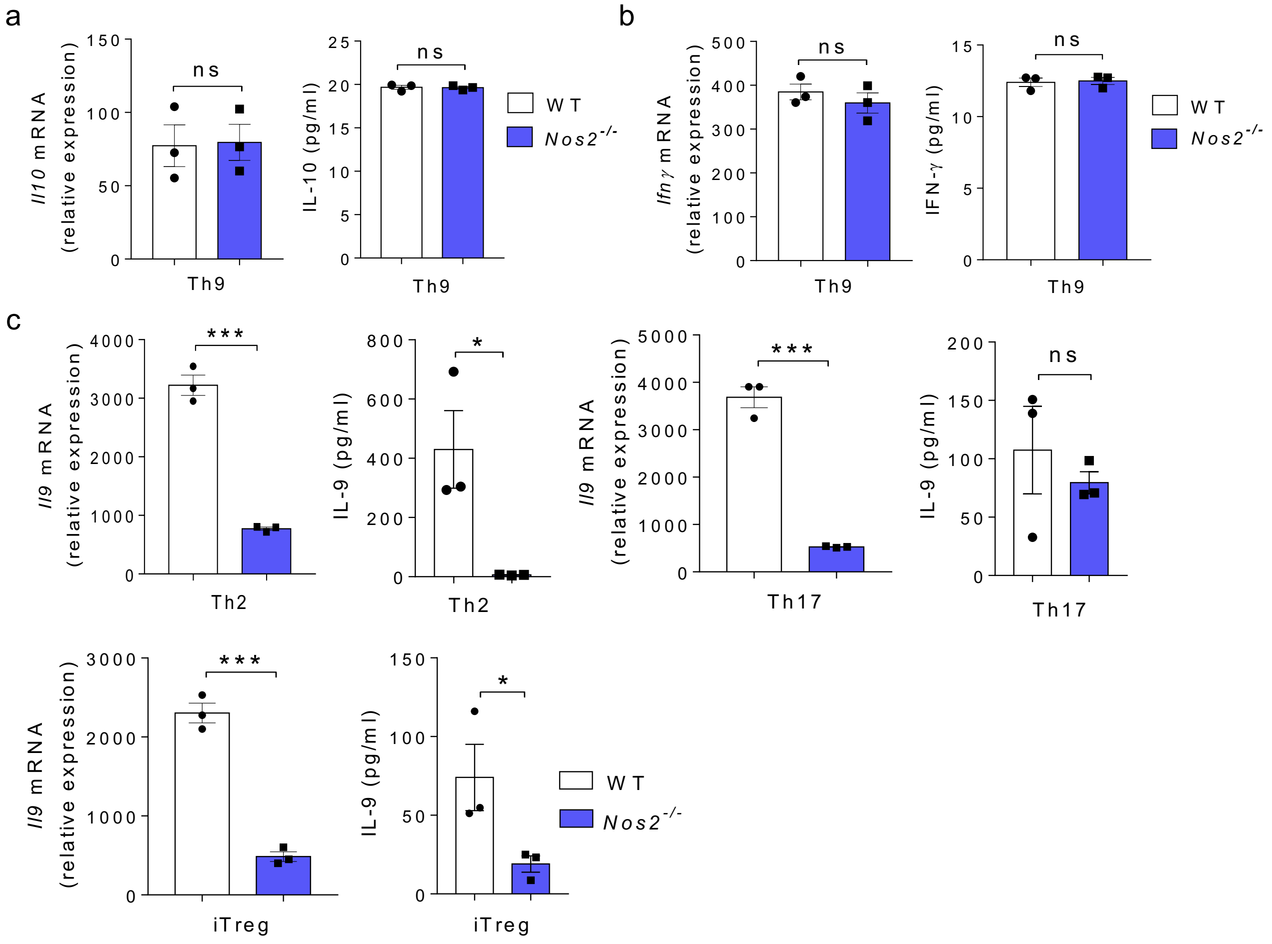

Supplementary Fig. 7. NO augments IL-9 induction in Th2, Th17, iTregs.

a,b. Naïve CD4^{+} T cells from WT and *Nos2*$^{-/-}$ mice were differentiated to Th9 followed by qPCR and ELISA for IL-10 and IFN-γ. Data are representative of mean ± SEM from three independent experiments. c. Naïve CD4^{+} T cells from WT and *Nos2*$^{-/-}$ mice were differentiated to Th2, Th17 and iTregs followed by qPCR and ELISA for IL-9. Data are representative of mean ± SEM from three independent experiments. a-c. *P < 0.0332, ***P < 0.0002, *P* = ns (not significant), using two-tailed unpaired student's t test.

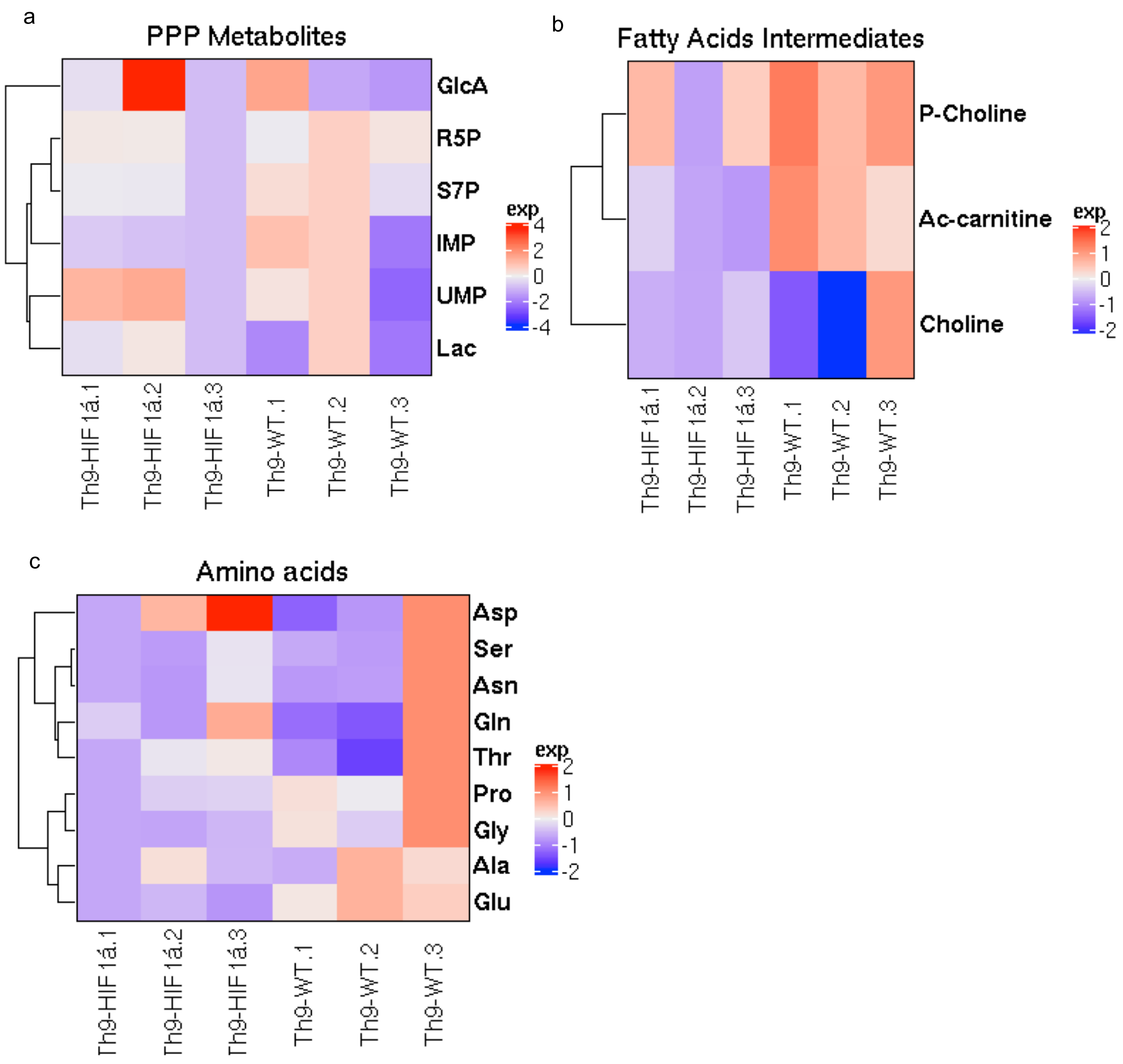

Supplementary Fig. 8. Metabolomics profiling of Th9 cells.

Naïve $CD4^+$ T cells from WT and *Hif1α*^{kd} mice were differentiated under Th9 polarizing conditions. Cell extracts were prepared and subjected to metabolomics profiling. Heat-maps showing (a) metabolites of Pentose Phosphate Pathway (PPP), (b) fatty acid intermediates and (c) amino acids.

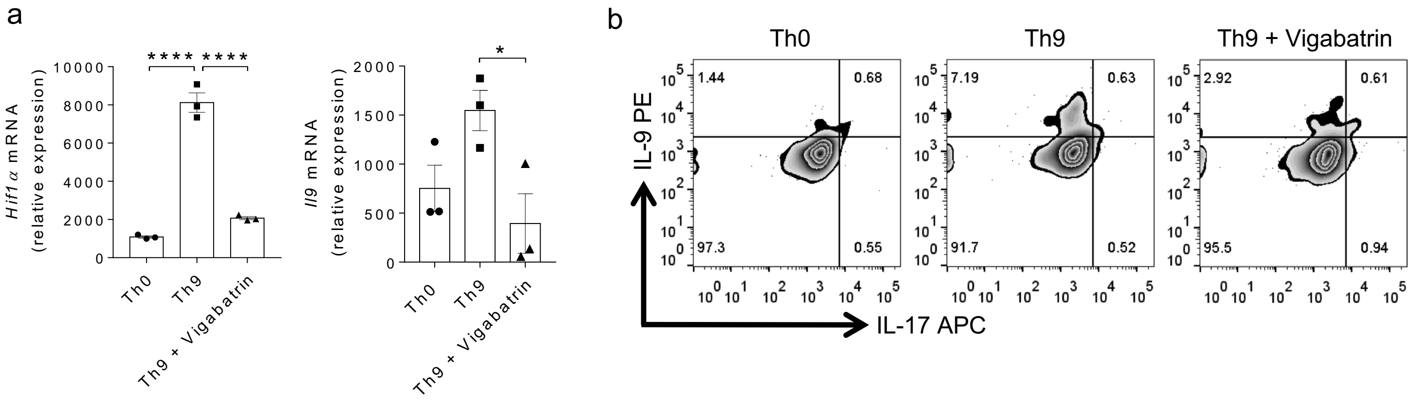

Supplementary Fig. 9. Vigabatrin inhibits HIF1α-mediated Th9 cell differentiation.

Naïve CD4$^+$ T cells from WT mice were differentiated under Th0 and Th9 polarizing conditions with or without 500 µM vigabatrin followed by a. qPCR analysis of *Hif1α* and *Il9* expression. Data are representative of mean ± SEM from three independent experiments. b. FACS analysis of intracellular IL-9 and IL-17. *P < 0.0332, ****P < 0.0001, using one-way ANOVA followed by Tukey's multiple comparison test.

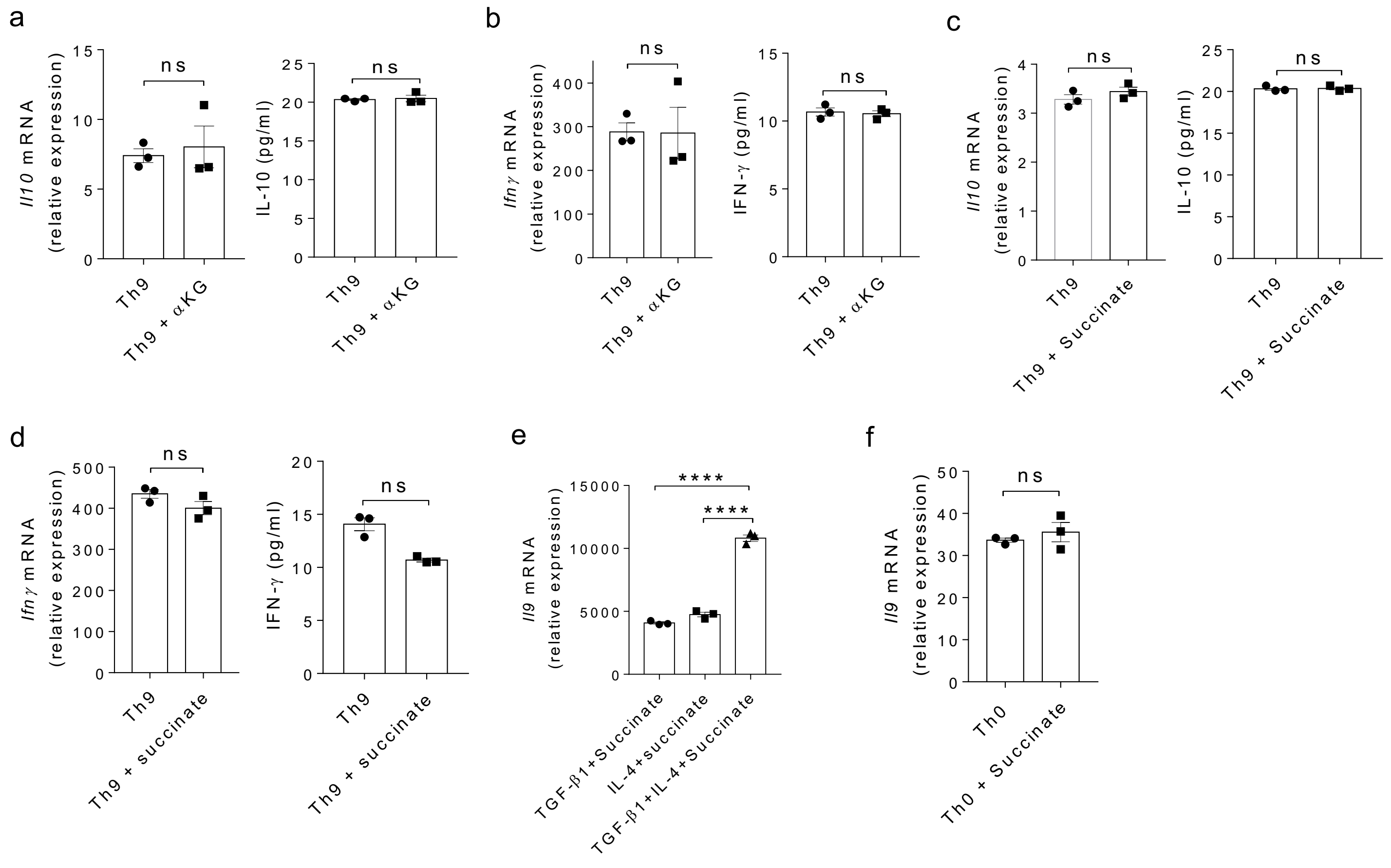

Supplementary Fig. 10. The effects of αKG and succinate are central to IL-9 and Th9 cells.

a,b. Naïve CD4$^+$ T cells from WT mice were differentiated under Th9 polarizing conditions with or without 1.0 mM αKG followed by qPCR and ELISA for IL-10 and IFN-γ. Data are representative of mean ± SEM from three independent experiments. c,d. Naïve CD4$^+$ T cells from WT mice were differentiated under Th9 polarizing conditions with or without 5.0 mM succinate followed by qPCR and ELISA for IL-10 and IFN-γ. Data are representative of mean ± SEM from three independent experiments e. Naïve CD4$^+$ T cells from WT mice were differentiated in the presence of TGF-β1 or IL-4 or TGF-β1+IL-4 with 5.0 mM succinate supplementation followed by qPCR analysis of *Il9* expression. Data are representative of mean ± SEM from three independent experiments. f. Naïve CD4$^+$ T cells from WT mice were cultured as Th0 with or without 5.0 mM succinate followed by qPCR analysis of *Il9* expression. Data are representative of mean ± SEM from three independent experiments. a,b,c,d,f. P = ns (not significant), using two-tailed unpaired student's t test. e. ****$P < 0.0001$, using one-way ANOVA followed by Tukey's multiple comparison test.

Supplementary Table 1. SYBR Primers for qPCR

Gene	Gene Sequence
**mβactin F**	5’-GATGTATGAAGGCTTTGGTC-3’
**mβactin R**	5’-TGTGCACTTTTATTGGTCTC-3’
**mHif1α F**	5’-CGATGACACAGAAACTGAAG-3’
**mHif1α R**	5’-GAAGGTAAAGGAGACATTGC-3’
**mIl9 F**	5’-CTGATGATTGTACCACACGTGC-3’
**mIl9 R**	5’-GCCTTTGCATCTCTGTCTTCTGG-3’
**mIl10 F**	5’-CAGGACTTTAAGGGTTACTTG-3’
**mIl10 R**	5’-ATTTTCACAGGGGAGAAATC-3’
**mIfnγ F**	5’-TGAGTATTGCCAAGTTTGAG-3’
**mIfnγ R**	5’-CTTATTGGGACAATCTCTTCC-3’
**mSpi1 F**	5’-CATGAGGTGAAATGTGAGAG-3’
**mSpi1 R**	5’-AGTTGGTTGAAATGGATCAC-3’
**mIrf4 F**	5’-ACGCTGCCCTCTTCAAGGCTT-3’
**mIrf4 R**	5’-TGGCTCCTCTCdCAATTCC-3’
**mGata3 F**	5’-TATTAACAGACCCCTGACTATG-3’
**mGata3 R**	5’-CACCTTTTTGCACTTTTTCG-3’
**mBatf F**	5’-AAAATGACAAGTCAACCCTG-3’
**mBatf R**	5’-TTAGAAAACTATCCACCCCC-3’
**mIrf1 F**	5’-TCTGTATAACCTACAGGTGTC-3’
**mIrf1 R**	5’- CAGACTGTTCAAAGAGCTTC -3’
**mEgfr F**	5'-TCTTCAAGGATGTGAAGTGTG-3’
**mEgfr R**	5'-TGTACGCTTTCGAACAATGT-3'
**mAreg F**	5’-GCCATTATGCAGCTGCTTTGGAGC-3’
**mAreg R**	5’-TGTTTTTCTTGGGCTTAATCACCT-3’
**mIl33r F**	5’- GGTTGCTCTGTTCTGGAGAGAT-3’
**mIl33r R**	5’-CTGCATCTTGCCCAGGTAAC-3’
**mNos2 F**	5’-TTTTGCATGACACTCTTCAC-3’
**mNos2 R**	5’-ACTGGTTGATGAACTCAATG-3’
**mEgln1 F**	5’-CCAAATGGAGATGGAAGATG-3’
**mEgln1 R**	5’-AGAATACCTCCACTTACCTTG-3’
**mTgfα F**	5’-AGCCAGAAGAAGCAAGCCATCACT-3’
**mTgfα R**	5’-CTCATTCTCGGTGTGGGTTAGCAA-3’
**mEgf F**	5’-AGATGAGTGTGTGCTGGCTAGATC-3’
**mEgf R**	5’-TCCGAGTCCTGTAGTAGTAAGTCC -3’
**mHbegf F**	5’-CTCCCACTGGATCCACAAAC-3’
**mHbegf R**	5’-GGCATGGGTCTCTCTTCTTC-3’
**hGapdh F**	5’-TGCACCACCAACTGCTTAGC-3’
**hGapdh R**	5’-GGCATGGACTGTGGTCATGAG-3’
**hIl9 F**	5’- GTGCCACTGCAGTGCTAATGT-3’
**hIl9 R**	5’-CTCTCACTGAAGCATGGTCTGG-3’
**hEgfr F**	5'-GCCAAGGCACGAGTAACAAGC-3'
**hEgfr R**	5'-AGGGCAATGAGGACATAACC-3'

Supplementary Table 2. SYBR Primers for ChIP-PCR

Gene	Gene Sequence
IL9P_HIF1α_site 1 RP	5'-TGCTGTAACCACTGAGCCAT-3'
IL9P_HIF1α_site 1 LP	5'-GTGACAGCAGACAAGAGCAG-3'
IL9P_HIF1α_site 2 RP	5'-ACTGCGGTGGGATATGTAGG-3'
IL9P_HIF1α_site 2 LP	5'-GAAGTGTGAGAGCAACGTGG-3'
IL9P_HIF1α_site 3 RP	5'-TGCCAGTGTATCAGTCAGGG-3'
IL9P_HIF1α_site 3 LP	5'-AGAATATGCATGCCTGGTGG-3'
IL9P_HIF1α_site 4 RP	5'-ACCACAGACCAGAGTTAGGC-3'
IL9P_HIF1α_site 4 LP	5'-CTCTCCCCTTCTACCTGCTG-3'
Nos2P_HIF1α_site 1 RP	5'-CATGCAAGGCCATCTCTTCC-3'
Nos2P_HIF1α_site 1 LP	5'-TCCGTGCCCAGAACAAAATC-3'
