# Supplementary material for: EGFR-HIF1α signaling positively regulates the differentiation of IL-9 producing T helper cells
Source: Nat Commun. 2021 Jun 1;12:3182. doi: 10.1038/s41467-021-23042-x (PMC8169867; doi:10.1038/s41467-021-23042-x)
Supplement: Supplementary file 2 — Description of Additional Supplementary Files [file 41467_2021_23042_MOESM2_ESM.pdf]

Description of Additional Supplementary Files

Supplementary Data 1: Coding for RNAseq analysis: The R code was for downstream analysis for which we have used this in-house script as indicated in supplementary data1. Using the in-house script, we further processed the data, then plotted the heatmap and PCA plot from RNAseq analysis.
