# Supplementary material for: EGFR-HIF1α signaling positively regulates the differentiation of IL-9 producing T helper cells
Source: Nat Commun. 2021 Jun 1;12:3182. doi: 10.1038/s41467-021-23042-x (PMC8169867; doi:10.1038/s41467-021-23042-x)
Supplement: Supplementary file 4 — Reporting summary [file 41467_2021_23042_MOESM4_ESM.pdf]

nature research

Corresponding author(s): Amit Awasthi

Last updated by author(s): 2021/02/20

Reporting Summary

Nature Research wishes to improve the reproducibility of the work that we publish. This form provides structure for consistency and transparency in reporting. For further information on Nature Research policies, see our Editorial Policies and the Editorial Policy Checklist.

Statistics

For all statistical analyses, confirm that the following items are present in the figure legend, table legend, main text, or Methods section.

n/a	Confirmed	
☐	☒	The exact sample size (*n*) for each experimental group/condition, given as a discrete number and unit of measurement
☐	☒	A statement on whether measurements were taken from distinct samples or whether the same sample was measured repeatedly
☐	☒	The statistical test(s) used AND whether they are one- or two-sided<br>*Only common tests should be described solely by name; describe more complex techniques in the Methods section.*
☐	☒	A description of all covariates tested
☐	☒	A description of any assumptions or corrections, such as tests of normality and adjustment for multiple comparisons
☐	☒	A full description of the statistical parameters including central tendency (e.g. means) or other basic estimates (e.g. regression coefficient) AND variation (e.g. standard deviation) or associated estimates of uncertainty (e.g. confidence intervals)
☐	☒	For null hypothesis testing, the test statistic (e.g. *F*, *t*, *r*) with confidence intervals, effect sizes, degrees of freedom and *P* value noted<br>*Give P values as exact values whenever suitable.*
☒	☐	For Bayesian analysis, information on the choice of priors and Markov chain Monte Carlo settings
☒	☐	For hierarchical and complex designs, identification of the appropriate level for tests and full reporting of outcomes
☒	☐	Estimates of effect sizes (e.g. Cohen's *d*, Pearson's *r*), indicating how they were calculated

Our web collection on statistics for biologists contains articles on many of the points above.

Software and code

Policy information about availability of computer code

Data collection

1. Flow cytometry data were collected on BD FACSCanto II with FACSDiva software version 8.0.2 (BD). Cells were sorted on BD FACS AriaIII with FACSDiva software version 8.0.2 (BD).
2. qRT-PCR data were collected on Fast 7500 Dx qPCR system (Applied Biosystems) and analyzed using SDS 2.1 software.
3. Metabolite detection data was collected and achieved with a Thermo Scientific Q Exactive mass spectrometer run with polarity switching in Full Scan mode with an m/z range of 65-975.
4. RNAseq data was collected on an Illumina HiSeq 2500.
5. Nanostring data was collected using nCounter system.

Data analysis

1. Flow cytometry data were analyzed on FlowJo version 10 (BD).
2. RNAseq analysis: The Quality check of the sequenced read were performed by FASTQC (version no. 0.11.9) and FASTX (version number 0.0.13) to remove the adaptor and unwanted low quality reads. Tophat2 and Bowtie2 packages were used to align the cleaned reads to the reference mouse genome (GRCm38). Subsequently, Htseq-count algorithm were used to measure gene expression from aligned reads. The read count-based gene expression data were normalized on the basis of library complexity and gene variation using the R package Cuffdiff. The normalized count data were compared between groups to identify differentially expressed genes. Genes were considered significantly differentially expressed if the P-value was <0.0001 FDR and absolute fold change cut-off was >2.
3. Ingenuity Pathway Analysis was done using IPA 8.0, Qiagen to identify the pathways that are significantly affected by differentially expressed genes.
4. The NanoString data were analyzed using nSolver Analysis software.
5. Mass spectrometry–based metabolomics analysis was done using in-house scripts in the statistical language R.
6. All the statistical analysis were performed on Prism version 7 (GraphPad).

For manuscripts utilizing custom algorithms or software that are central to the research but not yet described in published literature, software must be made available to editors and reviewers. We strongly encourage code deposition in a community repository (e.g. GitHub). See the Nature Research guidelines for submitting code & software for further information.

Data

Policy information about availability of data

All manuscripts must include a data availability statement. This statement should provide the following information, where applicable:

- Accession codes, unique identifiers, or web links for publicly available datasets
- A list of figures that have associated raw data
- A description of any restrictions on data availability

Sequence data that support the findings of this study have been deposited in GEO with the primary accession code, GSE163056. Publicly available data with accession code, GSE100634, were reanalyzed. The authors declare that all other data supporting the findings of this study are available within the article and its supplementary information files.

Field-specific reporting

Please select the one below that is the best fit for your research. If you are not sure, read the appropriate sections before making your selection.

☒ Life sciences ☐ Behavioural & social sciences ☐ Ecological, evolutionary & environmental sciences

For a reference copy of the document with all sections, see nature.com/documents/nr-reporting-summary-flat.pdf

Life sciences study design

All studies must disclose on these points even when the disclosure is negative.

Sample size	The sample size for in vivo and in vitro studies were determined based on our preliminary data. The sample size was selected to produce statistically relevant biological difference in the study.
Data exclusions	No data were excluded from the analyses.
Replication	All the experiments were replicated at least 3 times independently. For all in vitro and in vivo studies biological replicates were taken.
Randomization	Randomization of the mice were done based on their body weight or genotypes
Blinding	To evaluate unbiased disease phenotype blinding was done by 2 unbiased observers. Other data presented did not require the use of blinding. Data reported for mouse experiments were not subjective but rather based on quantitative analyses

Behavioural & social sciences study design

All studies must disclose on these points even when the disclosure is negative.

Study description	*Briefly describe the study type including whether data are quantitative, qualitative, or mixed-methods (e.g. qualitative cross-sectional, quantitative experimental, mixed-methods case study).*
Research sample	*State the research sample (e.g. Harvard university undergraduates, villagers in rural India) and provide relevant demographic information (e.g. age, sex) and indicate whether the sample is representative. Provide a rationale for the study sample chosen. For studies involving existing datasets, please describe the dataset and source.*
Sampling strategy	*Describe the sampling procedure (e.g. random, snowball, stratified, convenience). Describe the statistical methods that were used to predetermine sample size OR if no sample-size calculation was performed, describe how sample sizes were chosen and provide a rationale for why these sample sizes are sufficient. For qualitative data, please indicate whether data saturation was considered, and what criteria were used to decide that no further sampling was needed.*
Data collection	*Provide details about the data collection procedure, including the instruments or devices used to record the data (e.g. pen and paper, computer, eye tracker, video or audio equipment) whether anyone was present besides the participant(s) and the researcher, and whether the researcher was blind to experimental condition and/or the study hypothesis during data collection.*
Timing	*Indicate the start and stop dates of data collection. If there is a gap between collection periods, state the dates for each sample cohort.*
Data exclusions	*If no data were excluded from the analyses, state so OR if data were excluded, provide the exact number of exclusions and the rationale behind them, indicating whether exclusion criteria were pre-established.*
Non-participation	*State how many participants dropped out/declined participation and the reason(s) given OR provide response rate OR state that no participants dropped out/declined participation.*
Randomization	*If participants were not allocated into experimental groups, state so OR describe how participants were allocated to groups, and if allocation was not random, describe how covariates were controlled.*

Ecological, evolutionary & environmental sciences study design

All studies must disclose on these points even when the disclosure is negative.

Study description	*Briefly describe the study. For quantitative data include treatment factors and interactions, design structure (e.g. factorial, nested, hierarchical), nature and number of experimental units and replicates.*
Research sample	*Describe the research sample (e.g. a group of tagged Passer domesticus, all Stenocereus thurberi within Organ Pipe Cactus National Monument), and provide a rationale for the sample choice. When relevant, describe the organism taxa, source, sex, age range and any manipulations. State what population the sample is meant to represent when applicable. For studies involving existing datasets, describe the data and its source.*
Sampling strategy	*Note the sampling procedure. Describe the statistical methods that were used to predetermine sample size OR if no sample-size calculation was performed, describe how sample sizes were chosen and provide a rationale for why these sample sizes are sufficient.*
Data collection	*Describe the data collection procedure, including who recorded the data and how.*
Timing and spatial scale	*Indicate the start and stop dates of data collection, noting the frequency and periodicity of sampling and providing a rationale for these choices. If there is a gap between collection periods, state the dates for each sample cohort. Specify the spatial scale from which the data are taken*
Data exclusions	*If no data were excluded from the analyses, state so OR if data were excluded, describe the exclusions and the rationale behind them, indicating whether exclusion criteria were pre-established.*
Reproducibility	*Describe the measures taken to verify the reproducibility of experimental findings. For each experiment, note whether any attempts to repeat the experiment failed OR state that all attempts to repeat the experiment were successful.*
Randomization	*Describe how samples/organisms/participants were allocated into groups. If allocation was not random, describe how covariates were controlled. If this is not relevant to your study, explain why.*
Blinding	*Describe the extent of blinding used during data acquisition and analysis. If blinding was not possible, describe why OR explain why blinding was not relevant to your study.*

Did the study involve field work? ☐ Yes ☐ No

Field work, collection and transport

Field conditions	*Describe the study conditions for field work, providing relevant parameters (e.g. temperature, rainfall).*
Location	*State the location of the sampling or experiment, providing relevant parameters (e.g. latitude and longitude, elevation, water depth).*
Access & import/export	*Describe the efforts you have made to access habitats and to collect and import/export your samples in a responsible manner and in compliance with local, national and international laws, noting any permits that were obtained (give the name of the issuing authority, the date of issue, and any identifying information).*
Disturbance	*Describe any disturbance caused by the study and how it was minimized.*

Reporting for specific materials, systems and methods

We require information from authors about some types of materials, experimental systems and methods used in many studies. Here, indicate whether each material, system or method listed is relevant to your study. If you are not sure if a list item applies to your research, read the appropriate section before selecting a response.

Materials & experimental systems

n/a	Involved in the study	
☐	☒	Antibodies
☐	☒	Eukaryotic cell lines
☒	☐	Palaeontology and archaeology
☐	☒	Animals and other organisms
☐	☒	Human research participants
☒	☐	Clinical data
☒	☐	Dual use research of concern

Methods

n/a	Involved in the study	
☒	☐	ChIP-seq
☐	☒	Flow cytometry
☒	☐	MRI-based neuroimaging

Antibodies

Antibodies used	anti-mouse CD4 [RM4-5; BioLegend (CD4 PerCP Cat # 100538, CD4PE Cat #100512, CD4 APC Cat #100516) 1:200] and anti-mouse CD8a PerCP (53-6.7; BioLegend Cat # 100731; 1:200) for mouse, anti-mouse CD62L APC (MEL-14; Biolegend cat no. 104412, 1:200), anti-mouse CD44 PE/Cy7 (IM7; Biolegend cat no. 103030; 1:200) and anti-mouse CD25 PE (3C7, Biolegend cat no. 101904, 1:200). anti-CD3 (2.0 μg/ml; 145-2C11; Bioxcell cat no. BE0001-1) and anti-CD28 (2.0 μg/ml; 37.51; Bioxcell; BE0015-1), anti-human CD4 Horizon V450 (RPA-T4; BD Biosciences Cat # 560345; 1:200), anti-human CD45RA PE/Cy7 (HI100; Biolegend cat no. 304126; 1:200) and anti-human CD25 PE (BC96; Biolegend cat no. 302606; 1:200) , anti-human CD3 (10μg/ml; OKT-3; BioXcell cat no. BE0001-2) and soluble anti-human CD28 (3.0 ug/ml; BD, Bioscience cat no. 555725), anti-mouse IL-17A [TC11-18H10; BioLegend (IL-17A APC Cat # 506916, IL-17A PE/Cy7 Cat # 506904, IL-17A PE Cat # 506904, IL-17A Pacific Blue Cat # 506918, IL-17A FITC Cat # 506907); 1:200], anti-mouse IL-9 [RM9A4; BioLegend (IL-9 PE Cat # 514104, IL-9 PerCP/Cy5.5 Cat# 514112) 1:200], anti-mouse IFN-γ PE/Cy7 (XMG1.2; BioLegend Cat # 505816; 1:200) or anti-human IL-9 PerCP/Cy5.5 (MH9A4; BioLegend Cat #507610; 1:200) anti-HIF1α antibody (Abcam cat no. ab82832; 5ug per immunoprecipitation) or rabbit IgG ChIP grade antibody (Abcam cat no. ab46540, 1ug per immunopercipitation)
Validation	All antibodies were validated by the supplier (BioLegend, BioXcell, abcam) and were checked in the lab by comparing to the manufacturer's or inhouse results. Statement from BioLegend: BioLegend antibodies undergo an extensive series of testing to ensure quality at every step in the manufacturing process, as well as maintaining quality after the sale. Statement from Bio X Cell: Our InVivoPlus™antibodies feature all the great qualities of our InVivoMab™ antibodies. The InVivoPlus™ versions of our products are structurally and functionally identical to the InVivoMab™ versions with the added benefit of additional QC measures.

Eukaryotic cell lines

Policy information about cell lines

Cell line source(s)	B16-OVA was a kind gift from Lionel Apetoh (France) (Vegran et. al. 2014, Nature Immunology),HEK293T cell line was procured from American Type Culture Collection (ATCC).
Authentication	Tumor cell lines were authenticated by morphology, profileration in vitro and tumorogenicity and OVA specific functionality in viro. HEK293T cells authenticity was based on ATCC disclosure.
Mycoplasma contamination	All cell lines were tested negative for mycoplasma contamination.
Commonly misidentified lines (See ICLAC register)	No misidentified cell lines were used in this study.

Palaeontology and Archaeology

Specimen provenance	*Provide provenance information for specimens and describe permits that were obtained for the work (including the name of the issuing authority, the date of issue, and any identifying information).*
Specimen deposition	*Indicate where the specimens have been deposited to permit free access by other researchers.*
Dating methods	*If new dates are provided, describe how they were obtained (e.g. collection, storage, sample pretreatment and measurement), where they were obtained (i.e. lab name), the calibration program and the protocol for quality assurance OR state that no new dates are provided.*

☐ Tick this box to confirm that the raw and calibrated dates are available in the paper or in Supplementary Information.

Ethics oversight	*Identify the organization(s) that approved or provided guidance on the study protocol, OR state that no ethical approval or guidance was required and explain why not.*

Note that full information on the approval of the study protocol must also be provided in the manuscript.

Animals and other organisms

Policy information about studies involving animals; ARRIVE guidelines recommended for reporting animal research

Laboratory animals	C57BL/6 (#00064), OT-II TCR (#004194) and Nos2-/- (#002596), Egfrflox/floxXCd4cre mice were provided by D.M.W. Zaiss, Egfrflox/floxXCd4cre mice were performed at the University of Edinburgh, Areg-/- mice were provided by Fiona Powrie and Phd2kd and Hif1α kd mice were provided by Chris W. Pugh respectively. All animals used in the study were 6-12 weeks old and mixed gender. Laboratory animals were housed at institutional animal house facility maintained between 19 to 26°C ambient temperature with 30–70% humidity and 14 h light and 10 h dark cycle. All animals procedures were performed in laminar flow hoods.
Wild animals	Wild animals were not used in the study. Only inbred mice were used as described in the methods section of the manuscript
Field-collected samples	No field collected samples were used in the study.
Ethics oversight	The experiments on Egfrflox/floxXCd4cre mice were performed at the University of Edinburgh in accordance with university ethical guidelines. The experiments on Areg-/-, Phd2kd and Hif1αkd were performed at Kennedy Institute of Rheumatology, University of Oxford, United Kingdom in accordance to the institutional ethical guidelines. The samples were further shipped on dry ice to THSTI,

India for performing further assays and analysis. All animal experiments at THSTI were performed in accordance to the THSTI Animal Ethical guidelines.

Note that full information on the approval of the study protocol must also be provided in the manuscript.

Human research participants

Policy information about studies involving human research participants

Population characteristics	All the participants were aged between 25-35 years with no past history of disease or illness. Both male and female participants were included with their prior consent and approval.
Recruitment	Volunteers were well informed and their written consent was taken prior to sample collection. All the participants were healthy with no history of disease or illness. Participants were selected in randomly and in an unbiased manner.
Ethics oversight	THSTI Institutional human ethics committee approval was taken for the study involving PBMCs

Note that full information on the approval of the study protocol must also be provided in the manuscript.

Clinical data

Policy information about clinical studies

All manuscripts should comply with the ICMJEguidelines for publication of clinical research and a completedCONSORT checklist must be included with all submissions.

Clinical trial registration	*Provide the trial registration number from ClinicalTrials.gov or an equivalent agency.*
Study protocol	*Note where the full trial protocol can be accessed OR if not available, explain why.*
Data collection	*Describe the settings and locales of data collection, noting the time periods of recruitment and data collection.*
Outcomes	*Describe how you pre-defined primary and secondary outcome measures and how you assessed these measures.*

Dual use research of concern

Policy information about dual use research of concern

Hazards

Could the accidental, deliberate or reckless misuse of agents or technologies generated in the work, or the application of information presented in the manuscript, pose a threat to:

No	Yes	
☒	☐	Public health
☒	☐	National security
☒	☐	Crops and/or livestock
☒	☐	Ecosystems
☒	☐	Any other significant area

Experiments of concern

Does the work involve any of these experiments of concern:

No	Yes	
☒	☐	Demonstrate how to render a vaccine ineffective
☒	☐	Confer resistance to therapeutically useful antibiotics or antiviral agents
☒	☐	Enhance the virulence of a pathogen or render a nonpathogen virulent
☒	☐	Increase transmissibility of a pathogen
☒	☐	Alter the host range of a pathogen
☒	☐	Enable evasion of diagnostic/detection modalities
☒	☐	Enable the weaponization of a biological agent or toxin
☒	☐	Any other potentially harmful combination of experiments and agents

ChIP-seq

Data deposition

- [x] Confirm that both raw and final processed data have been deposited in a public database such as GEO.
- [x] Confirm that you have deposited or provided access to graph files (e.g. BED files) for the called peaks.

Data access links<br>*May remain private before publication.*	*For "Initial submission" or "Revised version" documents, provide reviewer access links. For your "Final submission" document, provide a link to the deposited data.*
Files in database submission	*Provide a list of all files available in the database submission.*
Genome browser session<br>(e.g. UCSC)	*Provide a link to an anonymized genome browser session for "Initial submission" and "Revised version" documents only, to enable peer review. Write "no longer applicable" for "Final submission" documents.*

Methodology

Replicates	*Describe the experimental replicates, specifying number, type and replicate agreement.*
Sequencing depth	*Describe the sequencing depth for each experiment, providing the total number of reads, uniquely mapped reads, length of reads and whether they were paired- or single-end.*
Antibodies	anti-HIF1α antibody (Abcam; ab1) or rabbit IgG ChIP grade antibody (Abcam; ab46540)
Peak calling parameters	*Specify the command line program and parameters used for read mapping and peak calling, including the ChIP, control and index files used.*
Data quality	*Describe the methods used to ensure data quality in full detail, including how many peaks are at FDR 5% and above 5-fold enrichment.*
Software	*Describe the software used to collect and analyze the ChIP-seq data. For custom code that has been deposited into a community repository, provide accession details.*

Flow Cytometry

Plots

Confirm that:

- [x] The axis labels state the marker and fluorochrome used (e.g. CD4-FITC).
- [x] The axis scales are clearly visible. Include numbers along axes only for bottom left plot of group (a 'group' is an analysis of identical markers).
- [x] All plots are contour plots with outliers or pseudocolor plots.
- [x] A numerical value for number of cells or percentage (with statistics) is provided.

Methodology

Sample preparation

6-12 weeks old WT mice were euthanized and spleen and lymph nodes were collected aseptically. Single cell suspensions from spleen and lymph nodes were prepared after lysing red blood cells using ACK lysis buffer. Cells were then stained with the cell surface antibodies- anti-mouse CD4 PerCP (RM4-5; BioLegend Cat # 100538; 1:200), anti-mouse CD62L APC (MEL-14; BioLegend Cat # 104412, 1:200), and anti-mouse CD25 PE (3C7, BioLegend Cat # 101904, 1:200). Cells were sorted on BD FACS Aria III with approximately >98% purity.

In Vitro human T helper cells differentiation
10 ml of peripheral blood was collected from healthy human volunteers after written informed consent in accordance with the approval of the institutional human ethics committee. Peripheral blood mononuclear cells (PBMCs) were isolated from whole blood using ficoll-paque based density gradient centrifugation and were then stained with the following cell surface fluorochrome-labelled antibodies: anti-human CD4 Horizon V450 (RPA-T4; BD Biosciences Cat # 560345; 1:200), anti-human CD45RA PE/Cy7 (HI100; BioLegend Cat # 304126; 1:200) and anti-human CD25 PE (BC96; BioLegend Cat # 302606; 1:200) and subjected to sorting on BD FACS Aria. Naïve CD4+ T cells (CD4+CD45RA+CD45RO-) were sorted on FACS BD FACS Aria III with >95% purity.

Intracellular cytokine staining and Flow cytometry
In vitro differentiated T cells were re-stimulated with PMA (phorbol 12-myristate13-acetate; 50 ng/ml; Sigma-Aldrich), ionomycin (1.0 µg/ml; Sigma-Aldrich) and monensin (GolgiStop, BD Biosciences Cat # 554724) for 6 hrs64. Cell surface staining was done for 15-20 min with anti-mouse CD4 [RM4-5; BioLegend (CD4 PerCP Cat # 100538, CD4PE Cat #100512, CD4 APC Cat #100516) 1:200] and anti-mouse CD8a PerCP (53-6.7; BioLegend Cat # 100731; 1:200) for mouse; and anti-human CD4 APC (OKT4, BioLegend Cat # 317416; 1:200) for human after live/dead marker staining respectively. For intracellular

	staining, cells were fixed in Cytofix solution and permeabilized with 1x Perm/Wash Buffer using kit (BD Biosciences Cat # 554714). Cells were then stained with anti-mouse IL-17A [TC11-18H10; BioLegend (IL-17A APC Cat # 506916, IL-17A PE/Cy7 Cat # 506904, IL-17A PE Cat # 506904, IL-17A Pacific Blue Cat # 506918, IL-17A FITC Cat # 506907); 1:200], anti-mouse IL-9 [RM9A4; BioLegend (IL-9 PE Cat # 514104, IL-9 PerCP/Cy5.5 Cat# 514112) 1:200], anti-mouse IFN-γ PE/Cy7 (XMG1.2; BioLegend Cat # 505816; 1:200) or anti-human IL-9 PerCP/Cy5.5 (MH9A4; BioLegend Cat #507610; 1:200) in Perm/Wash buffer. The cells were acquired using flow cytometry on FACSCantoII or with FACSDiva software version 8.0.2 (BD biosciences) and the results were analyzed with FlowJo software version 10 (Tree star).
Instrument	FACSCanto(BD biosciences) and BD FACS Aria III
Software	FACSDiva software version 8.0.2 (BD), FlowJo software 10 (Tree star)
Cell population abundance	Sorted mouse naive CD4+ T cell population with purity >98% and human naive CD4+ T cell population with purity >95% as determined in the post sort fraction.
Gating strategy	1. FACS sorting strategy of naive CD4+ T cell subsets from mice spleen and lymph nodes, (a) total splenocytes and lymph nodes cells were harvested, and then single cell suspension was prepared. Cells were gated on lymphocyte gate and size discrimination, doublet exclusion by double discrimination (FSC-H vs FSC-W and SSC-H vs SSC-W) were performed. CD4 +CD25- T cells were further sorted based on CD62L+ and CD44- gate as indicated. Purity of cells were tested in post-sort fraction. Alternatively, total splenocytes and lymph nodes cells were positively selected with anti-CD4 magnetic microbeads, and then total CD4+ T cells were further sorted using anti-CD62L antibody. Purity of cells were tested in post-sort fraction.<br><br>2. Highly purified naive CD4+CD45RA+ T cell subsets were obtained from healthy adult PBMC. Size discrimination, doublet exclusion by double discriminations method (FSC-H vs FSC-W and SSC-H vs SSC-W) were performed before gating them on CD4+ fraction, CD4+ naïve T cells were further sorted based on CD4+CD45RA+ CD45RO- gate. Purity of cells were tested in post-sort fraction.<br><br>3. Gating strategy for intracellular cytokine staining: Briefly activated cells were gated based on SSC and FCS, which were further gated on live cells. Live cells were then gated on CD4+ T cells in which intracellular cytokine staining was tested.

☒ Tick this box to confirm that a figure exemplifying the gating strategy is provided in the Supplementary Information.

Magnetic resonance imaging

Experimental design

Design type	*Indicate task or resting state; event-related or block design.*
Design specifications	*Specify the number of blocks, trials or experimental units per session and/or subject, and specify the length of each trial or block (if trials are blocked) and interval between trials.*
Behavioral performance measures	*State number and/or type of variables recorded (e.g. correct button press, response time) and what statistics were used to establish that the subjects were performing the task as expected (e.g. mean, range, and/or standard deviation across subjects).*

Acquisition

Imaging type(s)	*Specify: functional, structural, diffusion, perfusion.*
Field strength	*Specify in Tesla*
Sequence & imaging parameters	*Specify the pulse sequence type (gradient echo, spin echo, etc.), imaging type (EPI, spiral, etc.), field of view, matrix size, slice thickness, orientation and TE/TR/flip angle.*
Area of acquisition	*State whether a whole brain scan was used OR define the area of acquisition, describing how the region was determined.*

Diffusion MRI ☐ Used ☐ Not used

Preprocessing

Preprocessing software	*Provide detail on software version and revision number and on specific parameters (model/functions, brain extraction, segmentation, smoothing kernel size, etc.).*
Normalization	*If data were normalized/standardized, describe the approach(es): specify linear or non-linear and define image types used for transformation OR indicate that data were not normalized and explain rationale for lack of normalization.*
Normalization template	*Describe the template used for normalization/transformation, specifying subject space or group standardized space (e.g. original Talairach, MNI305, ICBM152) OR indicate that the data were not normalized.*

Noise and artifact removal

Describe your procedure(s) for artifact and structured noise removal, specifying motion parameters, tissue signals and physiological signals (heart rate, respiration).

Volume censoring

Define your software and/or method and criteria for volume censoring, and state the extent of such censoring.

Statistical modeling & inference

Model type and settings

Specify type (mass univariate, multivariate, RSA, predictive, etc.) and describe essential details of the model at the first and second levels (e.g. fixed, random or mixed effects; drift or auto-correlation).

Effect(s) tested

Define precise effect in terms of the task or stimulus conditions instead of psychological concepts and indicate whether ANOVA or factorial designs were used.

Specify type of analysis: ☐ Whole brain ☐ ROI-based ☐ Both

Statistic type for inference
(See Eklund et al. 2016)

Specify voxel-wise or cluster-wise and report all relevant parameters for cluster-wise methods.

Correction

Describe the type of correction and how it is obtained for multiple comparisons (e.g. FWE, FDR, permutation or Monte Carlo).

Models & analysis

n/a	Involved in the study
☐	☐ Functional and/or effective connectivity
☐	☐ Graph analysis
☐	☐ Multivariate modeling or predictive analysis

Functional and/or effective connectivity

Report the measures of dependence used and the model details (e.g. Pearson correlation, partial correlation, mutual information).

Graph analysis

Report the dependent variable and connectivity measure, specifying weighted graph or binarized graph, subject- or group-level, and the global and/or node summaries used (e.g. clustering coefficient, efficiency, etc.).

Multivariate modeling and predictive analysis

Specify independent variables, features extraction and dimension reduction, model, training and evaluation metrics.
